# IL-22 Binding Protein Controls IL-22–Driven Bleomycin-Induced Lung Injury

**DOI:** 10.1016/j.ajpath.2023.11.011

**Published:** 2024-03

**Authors:** Zhe Zhang, Mazvita B. Chakawa, Michelle Galeas-Pena, Joshua A. Frydman, Michaela J. Allen, MaryJane Jones, Derek Pociask

**Affiliations:** ∗ Department of Medicine, Pulmonary Diseases, Critical Care and Environmental Medicine, Tulane University School of Medicine, New Orleans, Louisiana; † Department of Immunology and Microbiology, Tulane University School of Medicine, New Orleans, Louisiana

## Abstract

The high mortality rates of acute lung injury and acute respiratory distress syndrome challenge the field to identify biomarkers and factors that can be exploited for therapeutic approaches. IL-22 is a cytokine that has antibacterial and reparative properties in the lung. However, it also can exacerbate inflammation and requires tight control by the extracellular inhibitory protein known as IL-22 binding protein (IL-22BP) (*Il22ra2*). This study showed the necessity of IL-22BP in controlling and preventing acute lung injury using IL-22BP knockout mice (*Il22ra2^−/−^*) in the bleomycin model of acute lung injury/acute respiratory distress syndrome. *Il22ra2^−/−^* mice had greater sensitivity (weight loss and death) and pulmonary inflammation in the acute phase (first 7 days) of the injury compared with wild-type C57Bl/6 controls. The inflammation was driven by excess IL-22 production, inducing the influx of pathogenic IL-17A^+^ γδ T cells to the lung. Interestingly, this inflammation was initiated in part by the noncanonical IL-22 signaling to macrophages, which express the IL-22 receptor (*Il22ra1*) *in vivo* after bleomycin challenge. This study further showed that IL-22 receptor alpha-1^+^ macrophages can be stimulated by IL-22 to produce a number of IL-17–inducing cytokines such as IL-1β, IL-6, and transforming growth factor-β1. Together, the results suggest that IL-22BP prevents IL-22 signaling to macrophages and reduces bleomycin-mediated lung injury.

## Graphical abstract

**Figure:**
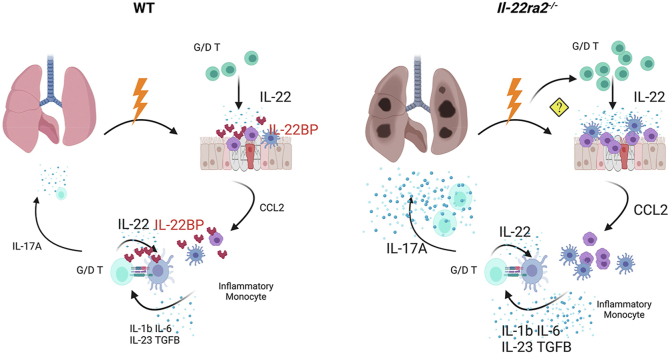

Acute lung injury (ALI) and subsequent acute respiratory distress syndrome (ARDS) account for approximately 10% to 15% of all patients admitted into a hospital’s intensive care unit. Before coronavirus disease-2019, ARDS affected approximately 190,000 people in the United States per year,^1^ and more than 3 million worldwide.^2^ Although treatment options have improved since the first descriptions of the disease in 1969, mortality still remains high (30% to 40% in most studies)^2^ and survivors often have diminished quality of life that lasts for months to years.^2^, ^3^, ^4^ Most of the persistent pulmonary dysfunction manifests as restrictive lung physiology^5^ resulting from sustained and often progressive fibrotic changes in the lung.^2^^,^^5^ The current significant increase of ARDS resulting from the coronavirus disease-2019 pandemic emphasizes a need to improve not only survival, but to reduce the long-term impact of ALI.

ALI manifests as a sudden and severe respiratory failure resulting from injury and inflammation in the lungs. The causes can be direct injury to the lung, as in the case of viral or bacterial infection, aspiration, or inhalation of harmful substances. ALI also can have indirect causes such as sepsis, severe trauma, and reperfusion injury.^6^^,^^7^ These disparate causes can make agreement of the appropriate models somewhat challenging. One model that has been well characterized is the bleomycin model of lung injury. Bleomycin is a glycopeptide antibiotic that induces oxidative stress, leading to epithelial and endothelial injury and subsequent inflammation, which exacerbates the injury.^8^, ^9^, ^10^ The inflammatory response has been the focus of many studies. It is thought that studying the immune response can lead to therapies that prevent mortality and progression to the fibrotic phases of the disease.

IL-22 is a cytokine that has shown much promise in terms of resolving lung infection and injury.^11^ This IL-10 family cytokine is secreted by T helper 17 cells, T helper 22 cells, γδ T cells, natural killer cells, natural killer T cells, innate lymphoid, and lymphoid-tissue inducer cells.^12^^,^^13^ IL-22 signals through the heterodimeric membrane–receptor complex of IL-22 receptor alpha-1/IL-10 receptor beta. In the lung, the receptor complex is expressed primarily on epithelial cells along the airways^14^ and is up-regulated in the parenchyma during infection and injury.^14^^,^^15^ IL-22 is critical for host defense against bacterial^16^, ^17^, ^18^ and viral pathogens,^14^^,^^16^^,^^19^, ^20^, ^21^ as well as important for maintaining tight junctions and promoting epithelial repair.^19^ However, IL-22 also can be deleterious, depending on its expression with other cytokines such as IL-17A,^22^ which has been implicated in lung cancer,^23^^,^^24^ emphysema,^25^, ^26^, ^27^ and allergic diseases.^28^, ^29^, ^30^ Therefore, it is important to understand the context of IL-22 expression and how it is controlled during a pulmonary insult.

IL-22 binding protein (IL-22BP) is a soluble receptor that efficiently binds to IL-22 and prevents signaling through the IL-22–receptor complex.^31^, ^32^, ^33^, ^34^ This inhibition has been proposed to act as a rheostat, controlling the concentration of IL-22 and reducing the potentially negative synergy with other cytokines such as IL-17A.^17^ It is integral for regulating excessive IL-22 and protecting from gastrointestinal cancer, arthritis, and psoriasis.^35^, ^36^, ^37^, ^38^, ^39^ However, IL-22BP can also have unwanted effects and prevent beneficial IL-22 responses. Specifically, this study showed that knocking out *Il22ra2* reduces viral-induced pneumonia,^19^^,^^20^ suggesting the balance between IL-22 and IL-22BP is more complex than initially thought.

This study showed IL-22 as proinflammatory and IL-22BP to have a protective role in the bleomycin-mediated ALI model. It showed that IL-22BP knockout mice (*Il22ra2*^*−/−*^) instilled with bleomycin had a greater sensitivity (weight loss and death), pulmonary injury, and inflammation than wild-type (WT) mice. *IL22ra2*^*−/−*^ mice had significantly greater production of IL-22 and IL-17. Specifically, *IL22ra2*^*−/−*^ mice had a significantly greater number of IL-17A^+^ γδ T cells. These data suggest that IL-22 has proinflammatory effects through signaling to macrophages because bleomycin induced the production of the Il22ra1 subunit of the IL-22 receptor. Moreover, IL-22 stimulated the macrophages producing a number of pro–IL-17 cytokines such as IL-1β, IL-6, and transforming growth factor-β1 (TGF-β1). This is the first report indicating that IL-22 can signal through macrophages to induce the secretion of multiple inflammatory cytokines and chemokines, leading to pulmonary damage and inflammation in the bleomycin-mediated ALI model.

## Materials and Methods

### Mice

All mice were bred in-house. *Il22ra2*^*−/−*^ mice and *Il22*^*−/−*^ mice were on a C57BL/6 background and have been described previously.^19^^,^^40^^,^^41^ The IL-22BP reporter mouse was developed with an IRES-TdTomato inserted between the coding sequence of exon 5 and the 3′ untranslated regions. Wild-type C57BL/6 breeder mice were purchased initially from Jackson Labs (Bar Harbor, ME). Male age-matched (age, 6 to 8 weeks) mice were used in all experiments. All mice were housed in the pathogen-free facility at Tulane University School of Medicine. All animal experiments were performed based on the protocols approved by Tulane University's Institutional Animal Care and Use Committee.

### Oropharyngeal Administration of Bleomycin and Lipopolysaccharide

Pharmaceutical grade bleomycin (Meitheal Pharmaceuticals, Chicago, IL) was instilled by oropharyngeal aspiration as described previously.^42^ Briefly, mice were anesthetized lightly with isoflurane and hung by their incisors. The tongue was pulled, and the pharynx was visualized. Bleomycin (100 μL) diluted in sterile phosphate-buffered saline (0.0025 U/g of mouse body weight for 21 days and 0.003 U/g of mouse body weight for 4 days) was instilled into the WT and *Il22ra2*^*−/−*^ mice lungs. Lipopolysaccharide (LPS) (50 μg diluted in 50 μL phosphate-buffered saline) (MilliporeSigma, St. Louis, MO) was administered by oropharyngeal aspiration in WT and *Il22ra2*^*−/−*^ mice. Mice were taken down on day 2 and day 4 after LPS administration for further analysis.

### Analysis of Lung Pathology

Mice were sacrificed at the indicated time points by severing the abdominal aorta to exsanguinate them.^14^^,^^15^ Lungs were perfused by injection of 5 mL sterile phosphate-buffered saline into the right ventricle of the heart to flush the remaining blood out of the pulmonary blood vessels. Right lungs were tied off and snap-frozen in liquid nitrogen for further protein and gene expression analysis while the left lung was inflated and fixed with 10% neutral-buffered formalin (TLD, Dawsonville, GA) for histology analysis. After dehydration and paraffin embedding, the paraffin block was sectioned at 5 μm and stained with hematoxylin and eosin. Collagen deposition was determined using Masson's trichrome stain. The severity of the lung injury after bleomycin treatment was scored based on weight loss and blind scoring of the hematoxylin and eosin stain and Masson's trichrome stain. The scale was as follows: 0 = no obvious inflammation; 1 = minimal focal inflammation; 2 = multifocal, mild inflammation; 3 = diffuse, conspicuous coalescing inflammation; and 4 = severe parenchymal inflammation with no obvious airspaces. The numeric scoring was described previously.^19^

### Enzyme-Linked Immunosorbent Assay and Bio-Plex

Whole-lung protein was isolated from lung tissue using Tissue Extraction Reagent I (Invitrogen, Carlsbad, CA). Protein was supplemented with Halt protease inhibitor cocktail (Thermo Fisher Scientific, Waltham, MA) and stored at −80°C. TGF-β1 enzyme-linked immunosorbent assays (ELISAs) were performed using the Mouse TGF-β1 DuoSet ELISA (R&D Systems, Minneapolis, MN). IL-22 ELISAs were performed using the Mouse IL-22 ELISA MAX Deluxe Set (BioLegend, San Diego, CA). Cytokine and chemokine analyses were performed from the whole-lung protein and cell culture supernatant by applying the Luminex-based miliplex multiplex suspension cytokine array (MilliporeSigma, Burlington, MA). The data were analyzed using Bio-Plex Manager software version 6.1 (Bio-Rad, Hercules, CA). The Bio-Plex assay was described previously.^43^

### Bronchoalveolar Lavage Fluid and Lactate Dehydrogenase Assay

Bronchoalveolar lavage fluid was acquired by inflating lungs with 1 mL sterile phosphate-buffered saline and repeated twice. The cell collection from the bronchoalveolar lavage fluid was achieved by centrifugation. Cell numbers were counted by a Nexcelom cellometer (Nexcelom, Lawrence, MA). To measure the lung injury, the first milliliter of bronchoalveolar lavage fluid supernatant was used for total protein measurement and lactate dehydrogenase assay (Abcam, Cambridge, UK). Total protein was measured by the BCA Protein Assay Kit (Pierce Chemical, Rockford, IL). These two measurements were performed according to the manufacturer's instructions and read by the Benchmark Plus plate reader (Bio-Rad).

### Real-Time PCR

Lung tissue RNA was isolated after mechanical homogenization of the lung by TRIzol reagent (Invitrogen) according to the manufacturer's instructions. The cDNA was synthesized using the reverse-transcription kit (Thermo Fisher Scientific) according to the manufacturer's instructions. TaqMan Gene Expression master mix (Thermo Fisher Scientific) was performed with primers to determine the gene expression level. Relative expression was calculated between the target gene and the housekeeping gene glyceraldehyde 3-phosphate dehydrogenase (*Gapdh*). TaqMan Gene Expression primers (Thermo Fisher Scientific) were used to determine the levels of the following: *Gapdh* (4352339E), *Il22* (Mm01226722_g1), *Il22ra2* (Mm001192969_m1), *Il17a* (Mm00439618_m1), *Il1b* (Mm00434228_m1), *Il6* (Mm00446190_m1), *Ccl2* (Mm00441242_m1), *Cxcl10* (Mm00445235_m1), and *Tgfb1* (Mm01227699_m1).

### Immunofluorescent Staining

Paraffin-embedded lung tissues were sectioned (5 μm) and placed on glass slides. Slides were dehydrated by passing slides through xylene washes followed by 2 × 5-minute washes through ethanol dilutions (100%, 95%, 75%). Antigen retrieval was performed using sodium citrate (10 mmol/L, 0.05% Tween 20, pH 6.0) in a microwave oven, bringing the slides to boiling and then cooling for 15 to 20 minutes. Slides were blocked in 5% goat serum (MilliporeSigma). Primary antibodies F4/80 (Cell Signaling, Danvers, MA), γδ T (BioLegend), and IL-22Ra1 (R&D Systems) were incubated overnight at a dilution of 1:200. Secondary antibodies goat anti-rabbit 488 and goat anti-rat 555 (Invitrogen) were incubated at a dilution of 1:500 for 2 hours. DAPI was used for counterstaining the nucleus. The EVOS FL Auto Imaging System (Thermo Fisher Scientific) was used for analysis.

### Flow Cytometry and Cell Sorting

A single-cell suspension was performed as previously described.^19^ For cell surface staining, cells were resuspended in flow cytometry blocking buffer (BD Biosciences, San Jose, CA) for staining with CD45, CD4, CD8, Ly6G, Ly6C, CD11b, CD11c, CD24, CD64, F4/80, T-cell receptor (TCR) γδ, and TCR β (Table 1). For Annexin V staining, cells were resuspended in Annexin V staining buffer (BioLegend) with phycoerythrin (PE) Annexin V and Helix NP Green (BioLegend). For cytokine staining, single cells were plated on a 96-well plate with 1 × 10^6^ cells/well and cultured in RPMI 1640 medium (GIBCO, Thermo Fisher Scientific) with 10% fetal bovine serum (GIBCO, Thermo Fisher Scientific) and 1% penicillin/streptomycin (GIBCO, Thermo Fisher Scientific). These cells were stimulated for 6 hours in the presence of phorbol myristate acetate (500 ng/mL; MilliporeSigma), ionomycin (500 ng/mL; MilliporeSigma), and brefeldin A (10 μg/mL; MilliporeSigma). Then, the cells were fixed and permeabilized using a fixation/permeabilization kit (BD Biosciences) for intracellular staining of IL-17A and IL-22 (Table 1). Finally, the stained cells were analyzed with a BD cell flow cytometry analyzer (Franklin Lakes, NJ) and FlowJo software version 9 (Ashland, OR). For the sorting experiment, cells were sorted using a fluorescence-activated cell sorting machine and cultured in RPMI 1640 medium with 10% fetal bovine serum and 1% penicillin/streptomycin. Cells were incubated on a 96-well U plate with IL-22 (150 ng/mL; R&D Systems) for 24 hours. Supernatant was used for Bio-Plex analysis. Cells were collected for RNA isolation as described in Real-Time PCR.

**Table 1:** Flow Cytometry Antibody List

Antigen	Clone	Form	Vendor	Catalog number
Anti-CD45	30-F11	Violet 450	Tonbo Biosciences (San Diego, CA)	75-0451-U100
Anti–IL-17A	TC11-18H10.1	FITC	BioLegend	506907
Anti-TCR γ/δ	GL3	PerCP-Cy5.5	BioLegend	118118
Anti–IL-22	1HBPWSR	PE	Invitrogen	12-7221-82
Anti-CD4	RM4-5	redFluor 710	Tonbo Biosciences	80-0042-U100
Anti-CD4	RM4-6	PerCP-Cy5.5	Tonbo Biosciences	65-0042-U100
Anti-TCR β	H57-597	PE	Tonbo Biosciences	50-5961-U100
Anti-TCR γ/δ	GL3	APC	BioLegend	118116
Anti-CD11B	M1/70	Violet 450	Tonbo Biosciences	75-0112-U100
Anti-CD24	M1/69	Brilliant Violet 605	BioLegend	101827
Anti-CD11C	N418	PE	BioLegend	117307
Anti-CD45	30-F11	PE/Cy5	BioLegend	103109
Anti-CD64	X54-5/7.1	PE/Cy7	BioLegend	139313
Anti-F4/80	BM8.1	APC	Tonbo Biosciences	20-4801-U100
Anti–Ly-6G	RB6-8C5	redFluor 710	Tonbo Biosciences	80-5931-U100
Anti–Ly-6C	HK1.4	APC/Fire	BioLegend	128046
Anti–Ly-6G	1A8	Brilliant Violet 421	BioLegend	127628
Anti-CD8a	53-6.7	Brilliant Violet 605	BioLegend	100744
Annexin V		PE	BioLegend	640947
Helix NP Green		FITC	BioLegend	425303

APC, allophycocyanin; Cy, cyanine; FITC, fluorescein isothiocyanate; PE, phycoerythrin; PerCP, peridinin chlorophyl protein; TCR, T-cell receptor.

### Statistical Analysis

An unpaired *t*-test, log-rank Mantel-Cox test, and ordinary two-way analysis of variance were applied to analyze differences between groups. All results are presented as the means ± SEM. *P* values < 0.05 were considered statistically significant. All statistics were calculated using GraphPad Prism 9.2 for Mac OS X (GraphPad Software, San Diego, CA).

## Results

### *Il22ra2*^*−/−*^ Mice Display Increased Sensitivity to Bleomycin

**Figure 1:**
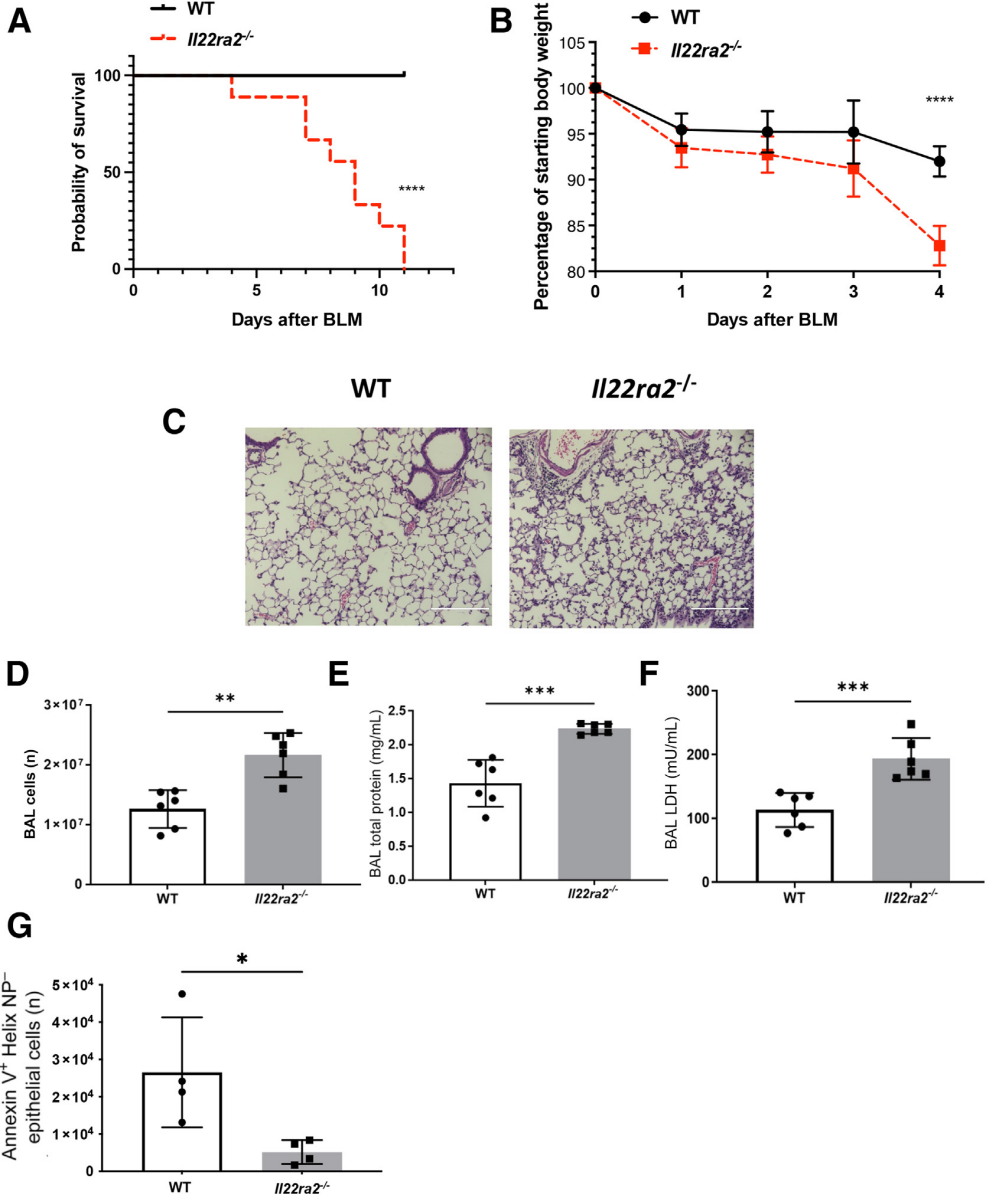
*Il22ra2*^*−/−*^ mice experience greater acute lung injury beginning on day 4. Wild-type (WT) and *Il22ra2*^*−/−*^ mice ages 6 to 8 weeks were treated with bleomycin (0.003 U/g) intratracheally. **A:** Survival of the WT and *Il22ra2*^*−/−*^ mice after bleomycin (0.003 U/g) treatment. **B:** Morbidity was measured by weight loss. **:** Histology of the lung from both groups in hematoxylin and eosin staining. **D:** Bronchoalveolar lavage (BAL) was collected on day 4. Cell numbers were counted by the Nexcelom cellometer. **E:** Total protein was measured by the BCA protein Assay kit. **F:** Lactate dehydrogenase was measured by the lactate dehydrogenase (LDH) assay. **G:** Apoptosis was measured from single-cell suspensions of whole lung by sorting epithelial cellular adhesion molecule (Epcam)^+^ Annexin V^+^ Helix NP Green^−^ cells. *n* = 9 mice/group (**A**); *n* = 6 mice/group (**B**). ∗∗∗∗*P* < 0.0001, log-rank Mantel-Cox test (**A**); ∗∗∗∗*P* < 0.0001, ordinary two-way analysis of variance (**B**); ∗*P* < 0.05, ∗∗*P* < 0.01, and ∗∗∗*P* < 0.001, unpaired *t*-test (**D**–**G**). Scale bar = 200 μm. BLM, bleomycin.

IL-22 is protective during influenza infection by improving the tight junction formation and decreasing lung inflammation.^19^ However, IL-22 also can have negative proinflammatory consequences, depending on the cytokine environment, necessitating its control by IL-22BP. Bleomycin injury may be one such environment where IL-22 requires this tight regulation because IL-22 production has been associated with increased inflammation.^44^ Therefore, to test the necessity of IL-22BP control of IL-22 during bleomycin challenge, *Il22ra2*^*−/−*^ mice were treated with bleomycin (0.003 U/g body weight by oropharyngeal aspiration). *Il22ra2*^*−/−*^ mice were extremely sensitive to bleomycin because they underwent rapid weight loss and total mortality within 10 days of administration (Figure 1A). Reducing the bleomycin dose to 0.0025 U/g led to increased survival. However, *Il22ra2*^*−/−*^ mice still displayed severe weight loss and severe histopathology at 21 days after administration (Supplemental Figure S1). Notably, C57Bl/6 WT mice displayed limited disease at this concentration of bleomycin. These data show that knocking out IL-22BP makes mice more sensitive to the harmful effects of bleomycin.

### *Il22ra2*^*−/−*^ Mice Experience Exacerbated Acute Lung Injury but Less Epithelial Apoptosis 4 Days after Bleomycin Administration

Bleomycin induces ALI within the first several days after administration.^45^ Given the rapid and severe weight loss seen in *Il22ra2*^*−/−*^ mice, these mice were hypothesized to undergo exacerbated ALI. To test this, mice were sacrificed 4 days after bleomycin administration because this was the time point at which weight loss began to diverge significantly from WT mice (Figure 1B). Hematoxylin and eosin staining showed the inflammatory changes in *Il22ra2*^*−/−*^ mice were interstitial, located at perivascular and peribronchiolar regions of the lung (Figure 1C). Bronchoalveolar lavage fluid analysis showed that *Il22ra2*^*−/−*^ mice had more injury as measured by total cell number (Figure 1D), total protein (Figure 1E), and lactate dehydrogenase levels (Figure 1F) compared with WT controls. However, analysis of epithelial cell apoptosis (annexin V^+^ Helix NP^–^) showed there was a mild but significant reduction in apoptosis in *Il22ra2*^*−/−*^ mice (Figure 1G). These data suggest that the phenotype was observed likely owing to exuberant inflammation.

**Figure 2:**
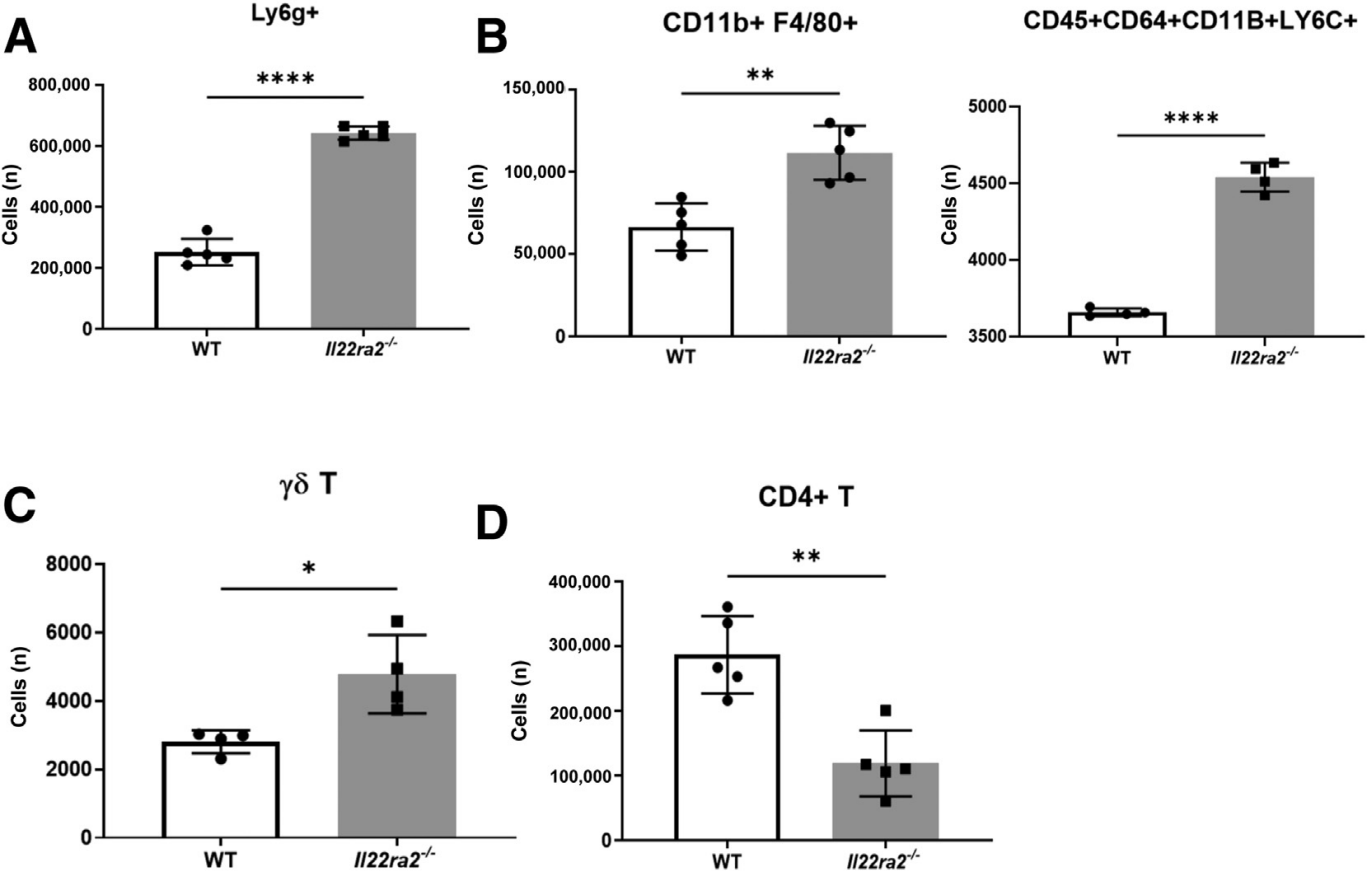
The character of the infiltrate on day 4 includes changes in specific myeloid, γδ cells, and CD4 T cells. Wild-type (WT) and *Il22ra2*^*−/−*^ mice ages 6 to 8 weeks were treated with bleomycin (0.003 U/g) intratracheally. Mice were euthanized on day 4. Lungs were collected and went through single-cell preparation. **A:** Cell numbers from flow cytometry showed CD45^+^Ly6g^+^ (neutrophils). **B:** CD11B^+^F4/80^+^, CD45^+^CD64^+^CD11B^+^Ly6C^+^ (inflammatory monocytes). **C:** γδ T cells. **D:** CD4 T cells. n = 4 mice/group. ∗*P* < 0.05, ∗∗*P* < 0.01, and ∗∗∗∗*P* < 0.0001, unpaired *t*-test.

To characterize the pulmonary inflammation, flow cytometric analysis was performed on a whole-lung, single-cell suspension and gated as in Supplemental Figure S2. Consistent with the bronchoalveolar lavage data, *Il22ra2*^*−/−*^ mice had significantly more neutrophils (Figure 2A) and inflammatory macrophages (CD45^+^CD11b^+^CD64^+^Ly6C^+^) (Figure 2B) than WT mice. At the same time, the number of γδ T cells increased significantly (Figure 2C), but CD4 T cell number decreased (Figure 2D).

### *Il22ra2*^*−/−*^ Mice Have Increased Inflammatory Cytokines and Chemokines in the Lung 4 Days after Bleomycin Administration

**Figure 3:**
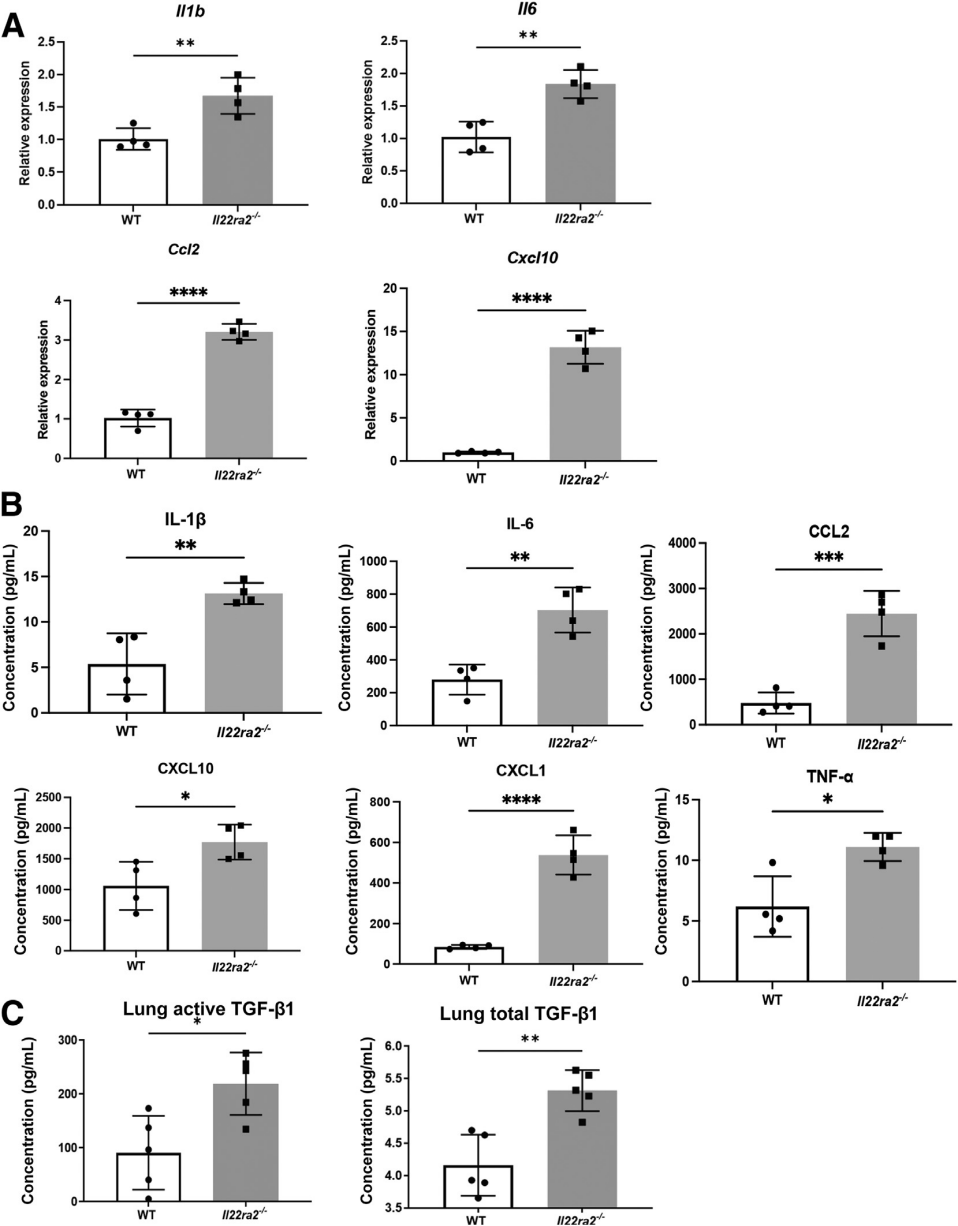
Higher inflammatory cytokines and chemokines in the *Il22ra2*^*−/−*^ mice after bleomycin treatment. Wild-type (WT) and *Il22ra2*^*−/−*^ mice ages 6 to 8 weeks were treated with bleomycin (0.003 U/g) intratracheally. Mice were euthanized on day 4. RNA and protein were isolated from whole lungs. **A:***Il1b*, *Il6*, *Ccl2*, and *Cxcl10* gene expression measured by quantitative RT-PCR. **B:** IL-1β, IL-6, CCL2, CXCL1, CXCL10, and tumor necrosis factor-α (TNF-α) protein level were detected by Bio-Plex. *n* = 4 mice/group. **C:** Active and total transforming growth factor-β1 (TGF-β1) levels were measured by the enzyme-linked immunosorbent assay. *n* = 5 mice/group. ∗*P* < 0.05, ∗∗*P* < 0.01, ∗∗∗*P* < 0.001, and ∗∗∗∗*P* < 0.0001, unpaired *t*-test.

The exacerbated inflammation observed in the *Il22ra2*^*−/−*^ mice led to the hypothesis that there would be increased inflammatory cytokines compared with WT mice. Quantitative PCR analysis showed significantly greater expression of *Il1b*, *Il6*, C*cl2*, and *Cxcl10* in the lungs of *Il22ra2*^*−/−*^ mice (Figure 3A). The Bio-Plex assay confirmed significantly higher protein concentrations of these inflammatory cytokines and chemokines in *Il22ra2*^*−/−*^ mice including IL-1β, IL-6, chemokine (C-C motif) ligand (CCL)2, CXCL10, CXCL1, tumor necrosis factor-α, leukemia inhibitory factor (LIF), CCL3, CCL4, and CXCL2 (Figure 3B and Supplemental Figure S3). Protein concentrations of both total and active TGF-β1 increased in *Il22ra2*^*−/−*^ mice (Figure 3C).

### γδ T cells Are the Predominant Source of IL-22 during Bleomycin Challenge

**Figure 4:**
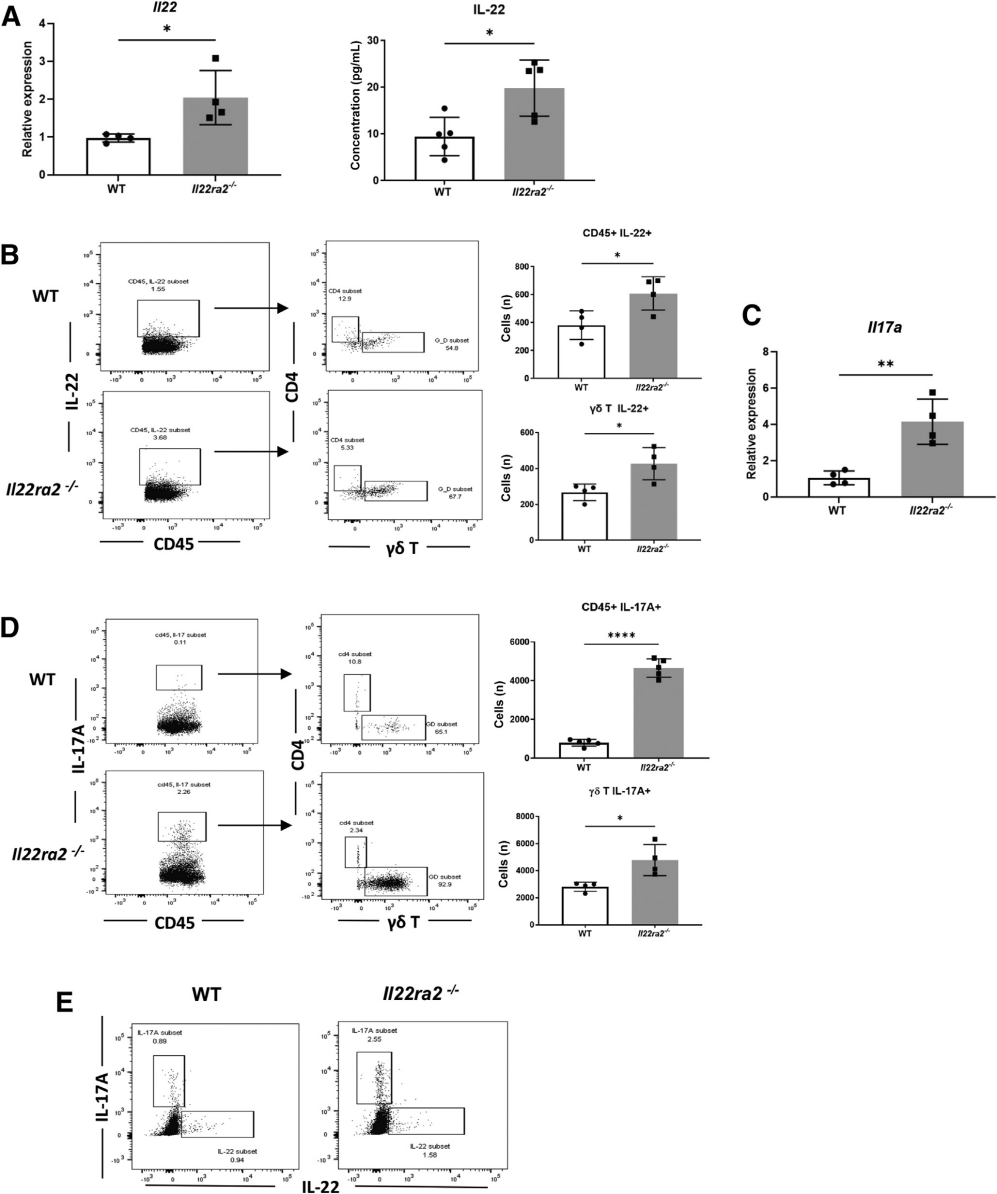
γδ T cells from *Il22ra2*^*−/−*^ mice display heightened production of IL-17A and IL-22. Wild-type (WT) and *Il22ra2*^*−/−*^ mice ages 6 to 8 weeks were treated with bleomycin (0.003 U/g) intratracheally. Mice were euthanized on day 4. RNA and protein were isolated from whole lungs. **A:***Il22* gene expression was measured by quantitative RT-PCR. IL-22 protein level was measured by enzyme-linked immunosorbent assay (ELISA). **B:** Flow cytometry representative dot plots show CD45^+^IL-22^+^ cells from the lungs of WT and *Il22ra2*^*−/−*^ mice. Then, the IL-22^+^ cells were gated on CD4 and γδ T cells. Bar graph shows the number of CD45^+^IL-22^+^ and γδ^+^IL-22^+^ cells in the lungs of WT and *Il22ra2*^*−/−*^ mice. **C:***Il17a* gene expression measured by quantitative RT-PCR. **D:** Flow cytometry representative dot plots show CD45^+^IL-17A^+^ cells from the lungs of WT and *Il22ra2*^*−/−*^ mice. Then, the IL-17A^+^ cells were gated on CD4 and γδ T cells. Bar graph shows the cell number of CD45^+^IL-17A^+^ and γδ^+^IL-17A^+^ cells in the lungs of WT and *Il22ra2*^*−/−*^ mice. **E:** Flow cytometry representative dot plots show IL-17A^+^ and IL-22^+^ cell populations in the lungs of WT and *Il22ra2*^*−/−*^ mice. *n* = 4 mice/group (A); *n* = 5 mice/group (**B**–**D**). ∗*P* < 0.05, ∗∗*P* < 0.01, and ∗∗∗∗*P* < 0.0001, unpaired *t*-test.

IL-22BP binding of IL-22 prevents its activity, as well as the ability to be detected by ELISA.^19^ ELISA results verified that *Il22ra2*^*−/−*^ mice had significantly higher concentrations of IL-22 protein. *Il22ra2*^*−/−*^ mice also had greater gene expression of *Il22* (Figure 4A). Flow cytometry analysis showed increased γδ T cells, which were the predominant IL-22–producing cell type 4 days after bleomycin challenge (Figure 4B).

### *Il22ra2*^*−/−*^ Mice Have Increased Numbers of IL-17A–Producing γδ T cells in the Lung

IL-17A is a cytokine associated with pulmonary fibrosis^46^, ^47^, ^48^ and has a pathologic synergy with IL-22.^44^ Given the severity of inflammation and increased expression of pro–IL-17 cytokines such as IL-1β, IL-6, and TGF-β1,^46^, ^47^, ^48^, ^49^, ^50^, ^51^ the expression and production of IL-17A was examined next. *Il22ra2*^*−/−*^ mice showed significantly greater gene expression of *Il17a* (Figure 4C). Flow cytometry analysis showed a significant increase in IL-17A^+^ γδ T cells in *Il22ra2*^*−/−*^ mice (Figure 4D). Interestingly, these IL17A^+^ γδ T cells were a different population from IL-22^+^ γδ T cells (Figure 4E). Importantly, there were similar percentages of γδ T cells in the lung between naïve WT and *Il22ra2*^*−/−*^ mice (Supplemental Figure S4).

### Macrophages and Dendritic Cells Are the Sources of IL-22BP during Bleomycin Challenge

**Figure 5:**
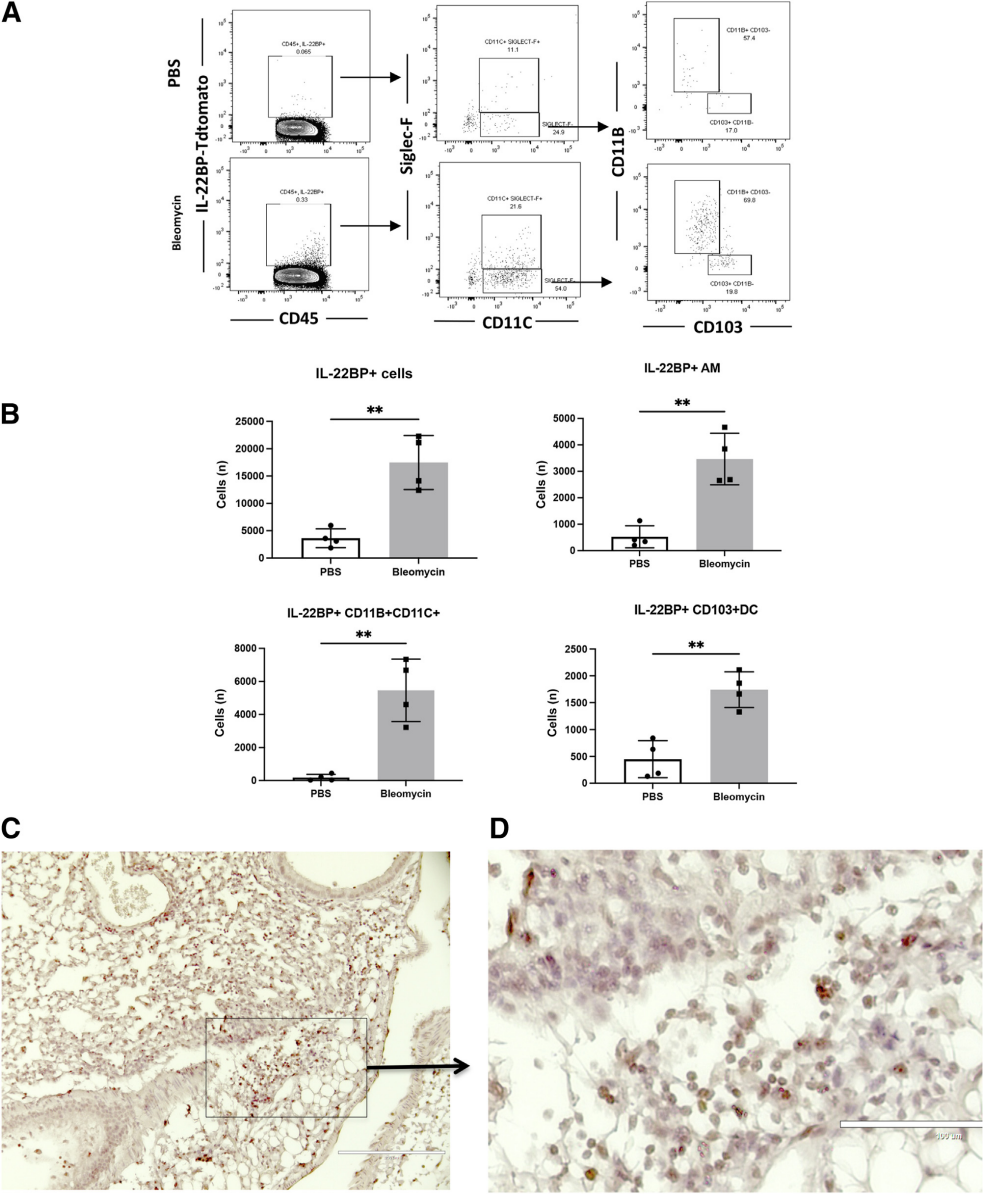
Macrophages and dendritic cells (DCs) are the main populations secreting IL-22 binding protein (IL-22BP) after bleomycin treatment. IL-22BP reporter mice ages 6 to 8 weeks were treated with bleomycin (0.003 U/g) and phosphate-buffered saline (PBS) intratracheally. Mice were euthanized on day 4. Lungs were collected and went through single-cell preparation. **A:** Flow cytometry representative dot plots showed CD45^+^IL-22BP^+^ (IL-22BP-Tdtomato^+^) cells in the lung of WT and *Il22ra2*^*−/−*^ mice after PBS or bleomycin treatment, which was gated further on Siglec-F^+^ versus CD11C^+^, then CD11C^+^Siglec-F^–^ cells were gated on CD11B^+^ versus CD103^+^. **B:** Cell numbers from flow cytometry showed IL-22BP^+^ alveolar macrophages (CD11C^+^Siglec-F^+^), CD103^+^ dendritic cells (CD11C^+^Siglec-F^–^CD103^+^), and CD11B^+^CD11C^+^ (CD11C^+^Siglec-F^–^CD11B^+^D103^–^) cells. **C:** Immunohistochemistry stanning showed IL-22BP-Tdtomato^+^ cells (IL-22BP^+^ cells) in the interstitial area of the lung after bleomycin treatment. **Boxed area** is enriched with IL-22BP ^+^ cells. **D:** Immunohistochemistry stanning shows Tdtomato^+^ cells (IL-22BP^+^ cells) enriched in the interstitial area of the lung after bleomycin treatment. *n* = 4 mice/group. ∗∗*P* < 0.01, unpaired *t*-test. Scale bars: 200 μm (**C**); 100 μm (**D**). AM, alveolar macrophage.

There are numerous difficulties finding antibodies to detect IL-22BP in mice. An IL-22BP reporter mouse was developed with an IRES-TdTomato inserted between the coding sequence of exon 5 and the 3′ untranslated region (Biocytogen, Wakefield, MA). Under naïve circumstances, very few IL-22BP–producing cells in the lung were detected (Figure 5, A and B). However, after bleomycin instillation, there was a significant increase in the total number of IL-22BP–producing cells (Figure 5, A and B). The primary source of IL-22BP are alveolar macrophages (CD11C^+^Siglec-F^+^), CD103^+^ dendritic cells (CD11C^+^Siglec-F^–^CD103^+^), and CD11B^+^CD11C^+^ (CD11C^+^Siglec-F^–^CD11B^+^D103^–^) cells (Figure 5, A and B). Immunohistochemistry staining of TdTomato showed that IL-22BP^+^ cells were in the perivascular space as well as the inflamed parenchyma (Figure 5, C and D).

### Macrophages Express IL-22Ra1 and Respond to IL-22 Activation

**Figure 6:**
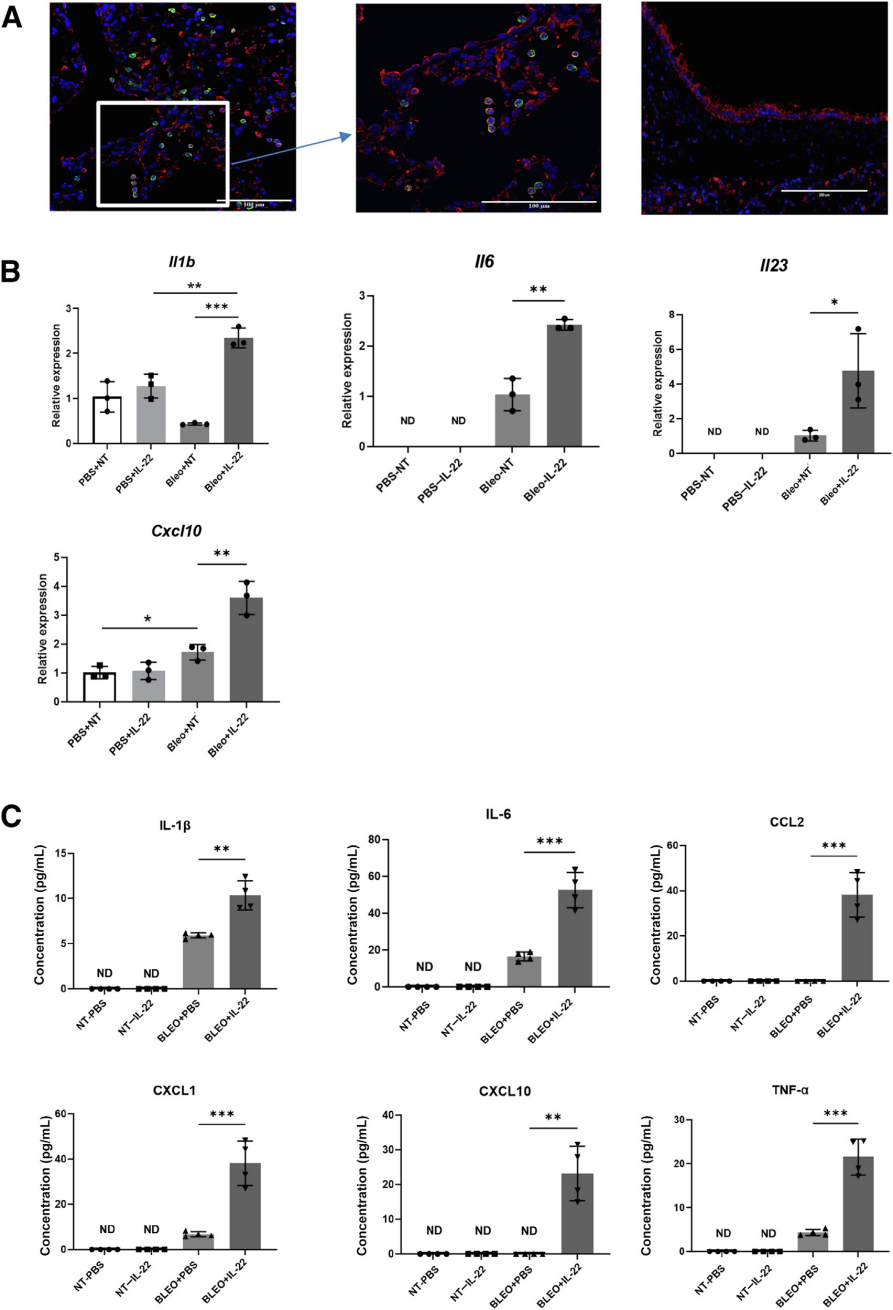
Macrophages express IL-22Ra1 and secrete more inflammatory cytokines and chemokines after IL-22 activation. **A:** Immunofluorescence staining of IL-22Ra1 (red), F4/80 (**green**), and nucleus (**blue**) in the bleomycin-treated lung parenchyma and airway. **Boxed area** is enriched with IL-22Ra1^+^ F4/80^+^ cells. **B:** Flow cytometry cell sorting was performed for lungs from bleomycin not treated (NT) and bleomycin-treated wild-type mice. The CD45^+^F4/80^+^ population was sorted and treated with phosphate-buffered saline (PBS) and IL-22 (30 ng/well) *in vitro*. *Il1b*, *Il6*, *Il23*, and *Cxcl10* expression were measured by quantitative RT-PCR. **C:** IL-1β, IL-6, CCL2, CXCL1, CXCL10, and tumor necrosis factor-α (TNF-α) protein levels were detected from the cell culture supernatant by Bio-Plex. *n* = 3 mice/group (**B**); *n* = 4 mice/group (**C**). ∗*P* < 0.05, ∗∗*P* < 0.01, and ∗∗∗*P* < 0.001, unpaired *t*-test. Scale bars: 100 μm (**A**, lung parenchyma) and 200 μm (**A**, airway). Bleo, bleomycin.

The receptor for IL-22 (IL-22Ra1) is generally found on epithelial surfaces in the naïve lung.^14^^,^^15^ However, several groups have identified it on macrophage populations.^52^^,^^53^ IL-22Ra1 immunofluorescence staining was performed on the lungs from the bleomycin model. IL-22Ra1 was found on epithelial cells as well as F4/80^+^ macrophages (Figure 6A). To prove that IL-22 can promote inflammatory cytokine and chemokine production from macrophages, cell sorting for F4/80^+^ macrophages from both naïve and bleomycin-treated WT mice was performed. Then, the sorted macrophages were treated with or without IL-22 *in vitro* for 24 hours. There was significant induction of the genes for *Il1b*, *Il6*, *Il23*, and *Cxcl10* from the IL-22–treated macrophages (Figure 6B). The Bio-Plex assay of the cell culture supernatant confirmed a higher protein level of certain inflammatory cytokines and chemokines in IL-22–treated macrophages, including IL-1β, IL-6, CCL2, CXCL1, CXCL10, tumor necrosis factor-α, CCL3, CCL4, and CXCL2 (Figure 6C and Supplemental Figure S5).

### Bleomycin-Induced Lung Injury Is IL-22 Dependent

**Figure 7:**
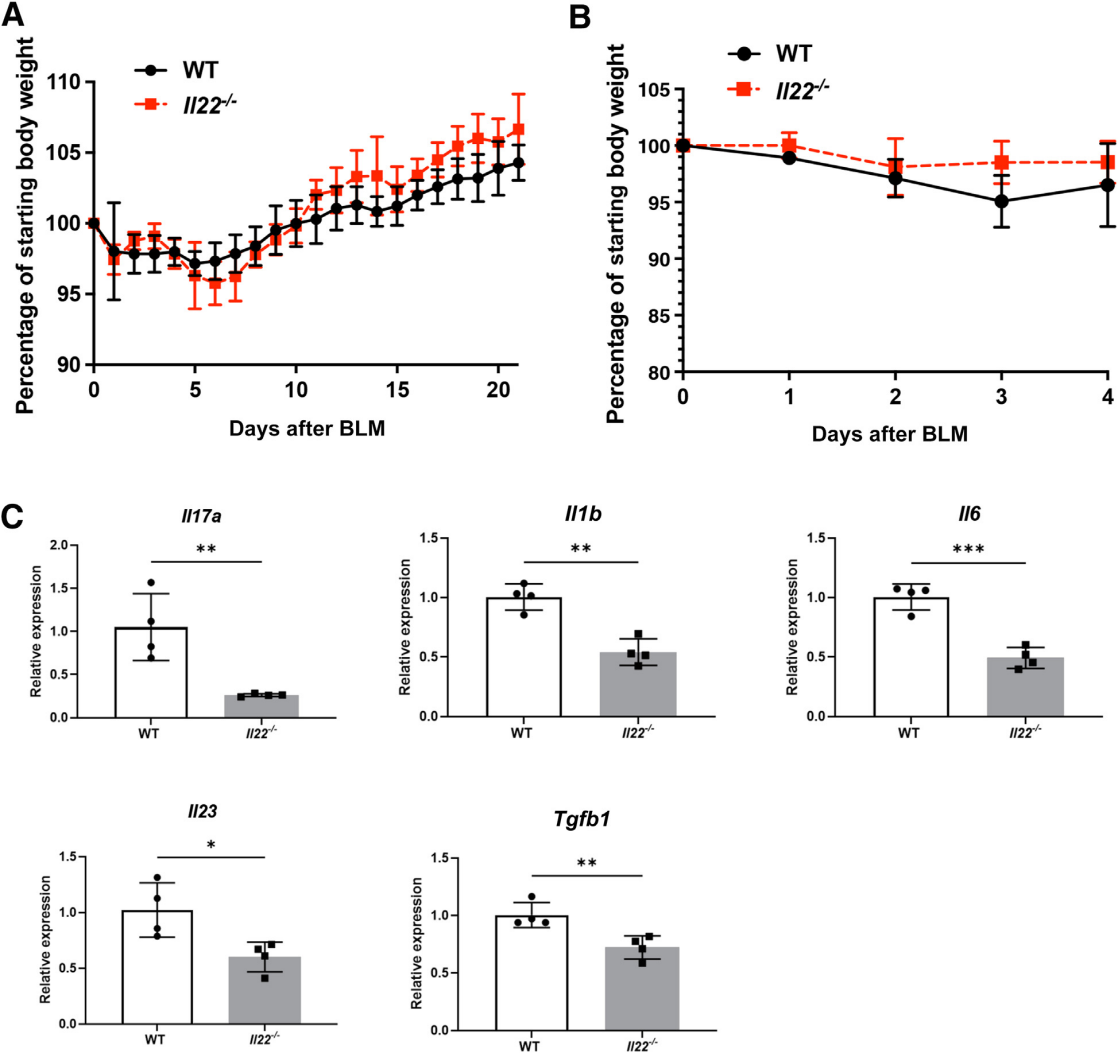
Bleomycin-induced lung injury is IL-22 dependent. **A:***Il22*^*−/−*^ mice and wild-type (WT) mice were treated with 0.0025 U/g bleomycin in a 21-day experiment. **B:***Il22*^*−/−*^ mice and WT mice were treated with 0.003 U/g bleomycin in a 4-day experiment. **C:***Il17a*, *Il1b*, *Il6*, *Il23*, and *Tgfb1* expressions were measured by quantitative RT-PCR. ∗*P* < 0.05, ∗∗*P* < 0.01, and ∗∗∗*P* < 0.001, unpaired *t*-test. BLM, bleomycin.

To show that bleomycin-induced lung injury is IL-22 dependent, both WT and *Il22*^*−/−*^ mice were treated with bleomycin. *Il22*^*−/−*^ mice did not display any significant differences in weight loss or mortality compared with WT mice (Figure 7, A and B). However, they did show a significant reduction in *Il17a*, *Il1b*, *Il6*, *Il23*, and *Tgfb1* gene expression as measured by quantitative PCR (Figure 7B), implying that bleomycin-induced lung injury is IL-22 dependent.

## Discussion

IL-22 has been described extensively as a cytokine that mediates cross-talk between the immune system and the pulmonary epithelium. This interaction leads to the induction of a number of peptides that are crucial for clearance and resolution of bacterial^16^, ^17^, ^18^ and fungal infections, as well as viral clearance^14^^,^^16^^,^^19^, ^20^, ^21^ and repair of the mucosal epithelium.^19^ Moreover, studies have suggested that production of IL-22BP is detrimental because *Il22ra2*^*−/−*^ display improved recovery in viral^19^, ^20^, ^21^ and bacterial^16^, ^17^, ^18^ infection models. These findings have led to questions regarding the necessity of IL-22BP in the lung. The data from the current study show that IL-22BP is required to prevent pathologic effects of IL-22 during a noninfectious injury. To our knowledge, this is the first report to show the critical necessity of controlling IL-22 in an acute lung injury model. During the first few days of bleomycin challenge, IL-22 is produced by γδ T cells. In the absence of IL-22BP, IL-22 leads to pathologic induction of IL-17, which exacerbates pulmonary neutrophilia and increases the risk of mortality. These findings show that controlling IL-22 is context-dependent.

There have been conflicting reports regarding the role of IL-22 in the bleomycin model. This is likely owing to the fact that some groups have focused on the initial ALI/ARDS phase of disease,^44^ whereas others have investigated the role of IL-22 in fibrosis.^54^, ^55^, ^56^, ^57^ This study showed that IL-22 is produced early after bleomycin challenge and, if it is unabated, as in *Il22ra2*^*−/−*^ mice, there is robust inflammation. These data align with the findings of Sonnenberg et al^44^ showing the proinflammatory nature of IL-22 in the initial stages of bleomycin injury. In these studies, IL-22 had a negative synergy with IL-17. It has been proposed that the role of IL-22 binding protein is to prevent this synergy. Previous data and the data from the current study support the idea that IL-22 is proinflammatory and requires control during the initial ALI stage of bleomycin challenge.

The heightened sensitivity to bleomycin precluded studying collagen deposition and fibrosis. Although it was possible to titer down bleomycin and show reduced mortality in the *Il22ra2*^*−/−*^ mice, the low levels of disease and inflammation in the WT mice precluded uncovering any subtleties of the model. Although the heightened sensitivity and exuberant inflammation seen here and in other studies^44^ suggest that IL-22 may promote fibrosis, there are conflicting data from other laboratories. Initial studies using a *Bacillus subtilis* model of hypersensitivity pneumonitis showed that IL-22 reduced inflammation and collagen deposition around the airways.^54^ However, this was a chronic infection model in which IL-22 is likely to have antibacterial effects. The data in noninfectious models were less straightforward because IL-22 was dispensable in a silicosis model.^58^ In the case of the bleomycin model, IL-22 ablation or knockout was associated with increased fibrosis^55^, ^56^, ^57^ and IL-22 prophylactic administration reduced epithelial to mesenchymal transition on epithelial cells^57^ and collagen production by fibroblasts.^56^ It is important to note that these studies were performed in the presence of IL-22BP, which reduced the potential pathogenic effects of IL-22 seen in these studies. Moreover, these studies showed the benefits of IL-22 acting upon the structural cells (epithelial cells and fibroblasts), which is similar to the findings in the current study that IL-22 mildly protected epithelial cells from apoptosis. In contrast, the data from this study show a pathogenic role for IL-22 when signaling to macrophages. These data suggest that the role for IL-22 may be more complex than initially thought and the inflammatory or protective nature of this cytokine may depend on the location in which it is produced.

ALI/ARDS is a disease that has many different causes and, likewise, many ways to model it. The most common model is administration of LPS to the lungs, which induces a rapid neutrophilic response through the Toll-like receptor 4 pathway.^59^ Although IL-22 administration has been shown to reduce inflammation in the LPS model of septic lung injury, this protection was seen only when given prophylactically.^60^ To our knowledge, there have not been any studies showing the endogenous role of IL-22 in this model. Supplemental Figure S6 shows that in the LPS model *Il22ra2*^*−/−*^ had mild yet significant increased inflammation compared with WT mice.

IL-22BP acts as a gatekeeper for IL-22 activity. It is expressed constitutively in the lung^19^^,^^61^ and must be modulated rapidly to allow or prevent IL-22 signaling. This is achieved through innate immune pattern recognition and often coincides with the production of IL-22.^62^ During infection, IL-22BP is down-regulated in response to Toll-like receptor 3^15^ and Toll-like receptor 4 stimuli,^40^ as well as DNA sensing by the inflammasome.^40^ Less is known about IL-22BP regulation during noninfectious injury such as bleomycin exposure. The current studies show that IL-22BP induction occurred early after exposure. IL-22BP induction can occur through the Toll-like receptor 2 retinoic acid pathway,^62^ which is induced directly by bleomycin.^63^ Bleomycin also induces excessive DNA damage, which is recognized by the cyclic GMP-AMP synthase (cGAS)–stimulator of interferon genes (STING) pathway,^64^^,^^65^ which also has been linked with IL-22BP.^66^ Further work is required to determine the importance of each of these potential pathways in promoting this protective factor.

The current data show a potential link between IL-22BP and acute lung injury, as well as pulmonary scarring and fibrosis. Although this is the first time this connection has been made in the lung, there are data suggesting a similar necessity for IL-22BP in the liver.^67^^,^^68^ In a model of acetaminophen toxicity, *Il22ra2*^*−/−*^ mice show heightened liver toxicity associated with IL-22 induction of CXCL10.^67^ In human studies, genetic variants of IL-22BP are associated with severe hepatic fibrosis in individuals infected with *Schistosoma japonicum*.^68^ These findings suggest that it is important to understand the context-specific requirement of the IL-22/IL-22BP axis in injury and repair.

The finding that γδ T cells were pathogenic in the model used in this study was somewhat surprising because it has been shown that γδ knockout (lacking the δ chain of the γδ T-cell receptor) mice have more pulmonary fibrosis.^42^^,^^54^^,^^69^ However, it should be noted that these studies are in lineage knockout mice in which other lymphocytes such as αβ T cells and innate lymphoid cells can compensate functionally for the absence of γδ T cells.^70^ The advent of the conditional ablation γδ mice (*Tcrd*-GDL mice)^70^^,^^71^ likely will offer more valuable insight into the role of γδ T cells.

γδ T cells are the predominant sources of both IL-22 and IL-17A, which exacerbate bleomycin-induced inflammation. This study showed that the IL-22^+^ γδ T cells are a separate cell group from IL-17A^+^ γδ T cells, suggesting different roles for different subtypes of γδ T cells. There are seven subtypes of γδ T cells in mice, and γδ17 cells mainly include Vγ6^+^ cells, Vγ4^+^ cells, and Vγ1^+^ cells.^70^^,^^72^^,^^73^ In a repeated *B. subtilis* infection-induced hypersensitivity pneumonitis model, Vγ6^+^Vδ1^+^ γδ T cells increase significantly and differentially secrete IL-17A and IL-22.^54^ These findings support the idea that different subsets of γδ T cells might have different functions during different phases of injury and repair. Although how γδ T cells are activated during bleomycin injury is unclear, it is known that they can recognize heat shock proteins 60 and 70, CD1C, and major histocompatibility complex I–related chains A and B.^42^^,^^74^, ^75^, ^76^, ^77^

IL-22 has been thought to signal only to epithelial cells; however, data from this study and other recent studies have shown that IL-22 also can signal directly to myeloid cells.^52^^,^^53^ Treerat et al showed that IL-22R–expressing macrophages have a protective role in controlling tuberculosis infection.^53^ Moreover, Trevejo-Nunez et al^52^ showed that IL-22R1^+^ macrophages are important for controlling pneumococcal infection. Furthermore, both mouse and human studies of chronic obstructive pulmonary disease (COPD) show that IL-22Ra1 expression by macrophages as well as increased IL-22 expression both are associated with exacerbated chronic obstructive pulmonary disease.^25^

The results presented here are critical in pointing to the unknown mechanism of ALI and improving the understanding of the role of IL-22/IL-22BP in this process, which may need to be taken into consideration for future treatment options.

## Disclosure Statement

None declared.

## Supplemental Data

Supplemental Figure S1 *Il22ra2*^*−/−*^ mice show higher morbidity and more lung injury than wild-type (WT) mice on day 21 after bleomycin treatment. WT and *Il22ra2*^*−/−*^ mice ages 6 to 8 weeks were treated with bleomycin at a lower dose (0.0025 U/g) intratracheally. **A:** Morbidity was measured by weight loss. **B:** Histology of the lungs from both groups on day 21 measured by hematoxylin and eosin (H&E) staining and Masson's trichrome staining. **C:** Lung pathology was scored blindly as described in *Materials and Methods*. *n* = 6 mice/group (**A**); *n* = 8 mice/group (**C**). ∗∗∗*P* < 0.001, unpaired *t*-test (**C**); ∗∗∗∗*P* < 0.0001, ordinary two-way analysis of variance (**A**). Scale bars = 200 μm. BLM, bleomycin.

Supplemental Figure S2 More γδ T cells, neutrophils, and inflammatory monocytes in the lungs of *Il22ra2*^*−/−*^ mice than in wild-type (WT) mice after bleomycin treatment. WT and *Il22ra2*^*−/−*^ mice ages 6 to 8 weeks were treated with bleomycin (0.003 U/g) intratracheally. Mice were euthanized on day 4. Lungs were collected and went through single-cell preparation. **A:** Gating strategy deployed to show all events, singlets, minus debris, and CD45^+^ populations. **B:** Flow cytometry representative dot plots showed CD45^+^Ly6g^+^ (neutrophils). **C:** CD45^+^CD64^+^CD11B^+^Ly6C^+^ (inflammatory monocytes). **D:** CD4 T cells and γδ T cells.

Supplemental Figure S3 Higher inflammatory cytokines and chemokines in the *Il22ra2*^*−/−*^ mice after bleomycin treatment. Wild-type (WT) and *Il22ra2*^*−/−*^ mice ages 6 to 8 weeks were treated with bleomycin (0.003 U/g) intratracheally. Mice were euthanized on day 4. Protein was isolated from whole lungs. Leukemia inhibitory factor (LIF), CCL3, CCL4, and CXCL2 protein levels were detected by Bio-Plex. *n* = 4 mice/group. ∗*P* < 0.05, ∗∗*P* < 0.01, and ∗∗∗∗*P* < 0.0001, unpaired *t*-test. ND, none determined.

Supplemental Figure S4 No difference in the CD4 and γδ T-cell populations between naïve WT and *Il22ra2*^*−/−*^ mice. Naïve wild-type and *Il22ra2*^*−/−*^ mice ages 6 to 8 weeks were used. Lungs were collected and went through single-cell preparation. Flow cytometry representative dot plots showed CD45^+^ cells, gated on T-cell receptor beta (TCRβ) versus γδ receptor and CD4 versus γδ receptor.

Supplemental Figure S5 Macrophages sorted from bleomycin-treated wild-type (WT) mice lungs secrete more inflammatory cytokines and chemokines after IL-22 activation. Flow cytometry cell sorting was performed on the lungs from bleomycin not treated (NT) and bleomycin-treated WT mice. The CD45^+^F4/80^+^ population was sorted and treated with phosphate-buffered saline (PBS) and IL-22 (30 ng/well) *in vitro*. **A:** Sorting strategy for the CD45^+^F4/80^+^ population. Cells first were gated on singlet, then scatter and live cells, and last on CD45^+^F4/80^+^. **B:** CCL3, CCL4, and CXCL2 protein levels detected from the cell culture supernatant by Bio-Plex. *n* = 4 mice/group. ∗∗∗*P* < 0.001, ∗∗∗∗*P* < 0.0001, unpaired *t*-test. BLEO, bleomycin; FSC-A, forward scatter area; FSC-H, forward scatter height; ND, not detected; SSC-A, side scatter area.

Supplemental Figure S6 Lipopolysaccharide (LPS) challenge of wild-type (WT) and Il22ra2^−/−^ mice show no differences in initial (48 hours) response, but delayed recovery of inflammation in *Il22ra2*^*−/−*^ mice. LPS (50 μg in 50 μL phosphate-buffered saline) was administered by tracheal aspiration in 6- to 8-week-old male WT and *Il22ra2*^*−/−*^ mice. There were no differences in weight loss or recovery in these mice. Mice were euthanized on days 2 and 4 after administration. **A** and **B:** Lungs were lavaged and analysis of bronchoalveolar lavage (BAL) showed no differences in lung (**A**) BAL cell number or (**B**) total BAL protein 48 hours after administration. **C** and **D:** Four days after administration, mild but significant delayed clearance of (**C**) BAL cell number and (**D**) total BAL protein was shown. ∗*P* < 0.05 as determined by unpaired *t*-test. ns, not significant.

## References

[1] Burnham E.L., Janssen W.J., Riches D.W., Moss M., Downey G.P. (2014). The fibroproliferative response in acute respiratory distress syndrome: mechanisms and clinical significance. Eur Respir J.

[2] Matthay M.A., Zemans R.L., Zimmerman G.A., Arabi Y.M., Beitler J.R., Mercat A., Herridge M., Randolph A.G., Calfee C.S. (2019). Acute respiratory distress syndrome. Nat Rev Dis Primers.

[3] Masclans J.R., Roca O., Munoz X., Pallisa E., Torres F., Rello J., Morell F. (2011). Quality of life, pulmonary function, and tomographic scan abnormalities after ARDS. Chest.

[4] Orme J., Romney J.S., Hopkins R.O., Pope D., Chan K.J., Thomsen G., Crapo R.O., Weaver L.K. (2003). Pulmonary function and health-related quality of life in survivors of acute respiratory distress syndrome. Am J Respir Crit Care Med.

[5] Burnham E.L., Hyzy R.C., Paine R., Coley C., Kelly A.M., Quint L.E., Lynch D., Janssen W.J., Moss M., Standiford T.J. (2013). Chest CT features are associated with poorer quality of life in acute lung injury survivors. Crit Care Med.

[6] Butt Y., Kurdowska A., Allen T.C. (2016). Acute lung injury: a clinical and molecular review. Arch Pathol Lab Med.

[7] Mowery N.T., Terzian W.T.H., Nelson A.C. (2020). Acute lung injury. Curr Probl Surg.

[8] Sleijfer S. (2001). Bleomycin-induced pneumonitis. Chest.

[9] Hay J., Shahzeidi S., Laurent G. (1991). Mechanisms of bleomycin-induced lung damage. Arch Toxicol.

[10] Hecht S.M. (2000). Bleomycin: new perspectives on the mechanism of action. J Nat Prod.

[11] Aujla S.J., Alcorn J.F. (2011). T(H)17 cells in asthma and inflammation. Biochim Biophys Acta.

[12] Rankin L.C., Girard-Madoux M.J., Seillet C., Mielke L.A., Kerdiles Y., Fenis A., Wieduwild E., Putoczki T., Mondot S., Lantz O., Demon D., Papenfuss A.T., Smyth G.K., Lamkanfi M., Carotta S., Renauld J.C., Shi W., Carpentier S., Soos T., Arendt C., Ugolini S., Huntington N.D., Belz G.T., Vivier E. (2016). Complementarity and redundancy of IL-22-producing innate lymphoid cells. Nat Immunol.

[13] Witte E., Witte K., Warszawska K., Sabat R., Wolk K. (2010). Interleukin-22: a cytokine produced by T, NK and NKT cell subsets, with importance in the innate immune defense and tissue protection. Cytokine Growth Factor Rev.

[14] Pociask D.A., Scheller E.V., Mandalapu S., McHugh K.J., Enelow R.I., Fattman C.L., Kolls J.K., Alcorn J.F. (2013). IL-22 is essential for lung epithelial repair following influenza infection. Am J Pathol.

[15] Hebert K.D., Mclaughlin N., Zhang Z., Cipriani A., Alcorn J.F., Pociask D.A. (2019). IL-22Ra1 is induced during influenza infection by direct and indirect TLR3 induction of STAT1. Respir Res.

[16] Ivanov S., Renneson J., Fontaine J., Barthelemy A., Paget C., Fernandez E.M., Blanc F., De Trez C., Van Maele L., Dumoutier L., Huerre M.R., Eberl G., Si-Tahar M., Gosset P., Renauld J.C., Sirard J.C., Faveeuw C., Trottein F. (2013). Interleukin-22 reduces lung inflammation during influenza A virus infection and protects against secondary bacterial infection. J Virol.

[17] Aujla S.J., Chan Y.R., Zheng M., Fei M., Askew D.J., Pociask D.A., Reinhart T.A., McAllister F., Edeal J., Gaus K., Husain S., Kreindler J.L., Dubin P.J., Pilewski J.M., Myerburg M.M., Mason C.A., Iwakura Y., Kolls J.K. (2008). IL-22 mediates mucosal host defense against gram-negative bacterial pneumonia. Nat Med.

[18] Xu X., Weiss I.D., Zhang H.H., Singh S.P., Wynn T.A., Wilson M.S., Farber J.M. (2014). Conventional NK cells can produce IL-22 and promote host defense in Klebsiella pneumoniae pneumonia. J Immunol.

[19] Hebert K.D., Mclaughlin N., Galeas-Pena M., Zhang Z., Eddens T., Govero A., Pilewski J.M., Kolls J.K., Pociask D.A. (2019). Targeting the IL-22/IL-22BP axis enhances tight junctions and reduces inflammation during influenza infection. Mucosal Immunol.

[20] Abood R.N., McHugh K.J., Rich H.E., Ortiz M.A., Tobin J.M., Ramanan K., Robinson K.M., Bomberger J.M., Kolls J.K., Manni M.L., Pociask D.A., Alcorn J.F. (2019). IL-22-binding protein exacerbates influenza, bacterial super-infection. Mucosal Immunol.

[21] Kumar P.A., Hu Y., Yamamoto Y., Hoe N.B., Wei T.S., Mu D., Sun Y., Joo L.S., Dagher R., Zielonka E.M., de Wang Y., Lim B., Chow V.T., Crum C.P., Xian W., McKeon F. (2011). Distal airway stem cells yield alveoli in vitro and during lung regeneration following H1N1 influenza infection. Cell.

[22] Sonnenberg G.F., Fouser L.A., Artis D. (2011). Border patrol: regulation of immunity, inflammation and tissue homeostasis at barrier surfaces by IL-22. Nat Immunol.

[23] Zhang W., Chen Y., Wei H., Zheng C., Sun R., Zhang J., Tian Z. (2008). Antiapoptotic activity of autocrine interleukin-22 and therapeutic effects of interleukin-22-small interfering RNA on human lung cancer xenografts. Clin Cancer Res.

[24] Kobold S., Volk S., Clauditz T., Kupper N.J., Minner S., Tufman A., Duwell P., Lindner M., Koch I., Heidegger S., Rothenfuer S., Schnurr M., Huber R.M., Wilczak W., Endres S. (2013). Interleukin-22 is frequently expressed in small- and large-cell lung cancer and promotes growth in chemotherapy-resistant cancer cells. J Thorac Oncol.

[25] Starkey M.R., Plank M.W., Casolari P., Papi A., Pavlidis S., Guo Y., Cameron G.J.M., Haw T.J., Tam A., Obiedat M., Donovan C., Hansbro N.G., Nguyen D.H., Nair P.M., Kim R.Y., Horvat J.C., Kaiko G.E., Durum S.K., Wark P.A., Sin D.D., Caramori G., Adcock I.M., Foster P.S., Hansbro P.M. (2019). IL-22 and its receptors are increased in human and experimental COPD and contribute to pathogenesis. Eur Respir J.

[26] Le Rouzic O., Pichavant M., Frealle E., Guillon A., Si-Tahar M., Gosset P. (2017). Th17 cytokines: novel potential therapeutic targets for COPD pathogenesis and exacerbations. Eur Respir J.

[27] Di Stefano A., Caramori G., Gnemmi I., Contoli M., Vicari C., Capelli A., Magno F., D'Anna S.E., Zanini A., Brun P., Casolari P., Chung K.F., Barnes P.J., Papi A., Adcock I., Balbi B. (2009). T helper type 17-related cytokine expression is increased in the bronchial mucosa of stable chronic obstructive pulmonary disease patients. Clin Exp Immunol.

[28] Massoud A.H., Charbonnier L.M., Lopez D., Pellegrini M., Phipatanakul W., Chatila T.A. (2016). An asthma-associated IL4R variant exacerbates airway inflammation by promoting conversion of regulatory T cells to TH17-like cells. Nat Med.

[29] Nakagome K., Imamura M., Kawahata K., Harada H., Okunishi K., Matsumoto T., Sasaki O., Tanaka R., Kano M.R., Chang H., Hanawa H., Miyazaki J., Yamamoto K., Dohi M. (2011). High expression of IL-22 suppresses antigen-induced immune responses and eosinophilic airway inflammation via an IL-10-associated mechanism. J Immunol.

[30] Pennino D., Bhavsar P.K., Effner R., Avitabile S., Venn P., Quaranta M., Marzaioli V., Cifuentes L., Durham S.R., Cavani A., Eyerich K., Chung K.F., Schmidt-Weber C.B., Eyerich S. (2013). IL-22 suppresses IFN-gamma-mediated lung inflammation in asthmatic patients. J Allergy Clin Immunol.

[31] Kotenko S.V., Izotova L.S., Mirochnitchenko O.V., Esterova E., Dickensheets H., Donnelly R.P., Pestka S. (2001). Identification, cloning, and characterization of a novel soluble receptor that binds IL-22 and neutralizes its activity. J Immunol.

[32] Wei C.C., Ho T.W., Liang W.G., Chen G.Y., Chang M.S. (2003). Cloning and characterization of mouse IL-22 binding protein. Genes Immun.

[33] Dumoutier L., Lejeune D., Colau D., Renauld J.C. (2001). Cloning and characterization of IL-22 binding protein, a natural antagonist of IL-10-related T cell-derived inducible factor/IL-22. J Immunol.

[34] Jones B.C., Logsdon N.J., Walter M.R. (2008). Structure of IL-22 bound to its high-affinity IL-22R1 chain. Structure.

[35] Eyerich S., Eyerich K., Pennino D., Carbone T., Nasorri F., Pallotta S., Cianfarani F., Odorisio T., Traidl-Hoffmann C., Behrendt H., Durham S.R., Schmidt-Weber C.B., Cavani A. (2009). Th22 cells represent a distinct human T cell subset involved in epidermal immunity and remodeling. J Clin Invest.

[36] Kagami S., Rizzo H.L., Lee J.J., Koguchi Y., Blauvelt A. (2010). Circulating Th17, Th22, and Th1 cells are increased in psoriasis. J Invest Dermatol.

[37] Kim K.W., Kim H.R., Park J.Y., Park J.S., Oh H.J., Woo Y.J., Park M.K., Cho M.L., Lee S.H. (2012). Interleukin-22 promotes osteoclastogenesis in rheumatoid arthritis through induction of RANKL in human synovial fibroblasts. Arthritis Rheum.

[38] Zhuang Y., Peng L.S., Zhao Y.L., Shi Y., Mao X.H., Guo G., Chen W., Liu X.F., Zhang J.Y., Liu T., Luo P., Yu P.W., Zou Q.M. (2012). Increased intratumoral IL-22-producing CD4(+) T cells and Th22 cells correlate with gastric cancer progression and predict poor patient survival. Cancer Immunol Immunother.

[39] Wu T., Cui L., Liang Z., Liu C., Liu Y., Li J. (2013). Elevated serum IL-22 levels correlate with chemoresistant condition of colorectal cancer. Clin Immunol.

[40] Huber S., Gagliani N., Zenewicz L.A., Huber F.J., Bosurgi L., Hu B., Hedl M., Zhang W., O'Connor W., Murphy A.J., Valenzuela D.M., Yancopoulos G.D., Booth C.J., Cho J.H., Ouyang W., Abraham C., Flavell R.A. (2012). IL-22BP is regulated by the inflammasome and modulates tumorigenesis in the intestine. Nature.

[41] Ahlfors H., Morrison P.J., Duarte J.H., Li Y., Biro J., Tolaini M., Di Meglio P., Potocnik A.J., Stockinger B. (2014). IL-22 fate reporter reveals origin and control of IL-22 production in homeostasis and infection. J Immunol.

[42] Pociask D.A., Chen K., Choi S.M., Oury T.D., Steele C., Kolls J.K. (2011). gammadelta T cells attenuate bleomycin-induced fibrosis through the production of CXCL10. Am J Pathol.

[43] Godwin M.S., Jones M., Blackburn J.P., Yu Z., Matalon S., Hastie A.T., Meyers D.A., Steele C. (2021). The chemokine CX3CL1/fractalkine regulates immunopathogenesis during fungal-associated allergic airway inflammation. Am J Physiol Lung Cell Mol Physiol.

[44] Sonnenberg G.F., Nair M.G., Kirn T.J., Zaph C., Fouser L.A., Artis D. (2010). Pathological versus protective functions of IL-22 in airway inflammation are regulated by IL-17A. J Exp Med.

[45] Della Latta V., Cecchettini A., Del Ry S., Morales M.A. (2015). Bleomycin in the setting of lung fibrosis induction: from biological mechanisms to counteractions. Pharmacol Res.

[46] Gasse P., Riteau N., Vacher R., Michel M.L., Fautrel A., di Padova F., Fick L., Charron S., Lagente V., Eberl G., Le Bert M., Quesniaux V.F., Huaux F., Leite-de-Moraes M., Ryffel B., Couillin I. (2011). IL-1 and IL-23 mediate early IL-17A production in pulmonary inflammation leading to late fibrosis. PLoS One.

[47] Mi S., Li Z., Yang H.Z., Liu H., Wang J.P., Ma Y.G., Wang X.X., Liu H.Z., Sun W., Hu Z.W. (2011). Blocking IL-17A promotes the resolution of pulmonary inflammation and fibrosis via TGF-beta1-dependent and -independent mechanisms. J Immunol.

[48] Wilson M.S., Madala S.K., Ramalingam T.R., Gochuico B.R., Rosas I.O., Cheever A.W., Wynn T.A. (2010). Bleomycin and IL-1beta-mediated pulmonary fibrosis is IL-17A dependent. J Exp Med.

[49] Willis B.C., Borok Z. (2007). TGF-beta-induced EMT: mechanisms and implications for fibrotic lung disease. Am J Physiol Lung Cell Mol Physiol.

[50] Chen T., Nie H., Gao X., Yang J., Pu J., Chen Z., Cui X., Wang Y., Wang H., Jia G. (2014). Epithelial-mesenchymal transition involved in pulmonary fibrosis induced by multi-walled carbon nanotubes via TGF-beta/Smad signaling pathway. Toxicol Lett.

[51] Hisatomi K., Mukae H., Sakamoto N., Ishimatsu Y., Kakugawa T., Hara S., Fujita H., Nakamichi S., Oku H., Urata Y., Kubota H., Nagata K., Kohno S. (2012). Pirfenidone inhibits TGF-beta1-induced over-expression of collagen type I and heat shock protein 47 in A549 cells. BMC Pulm Med.

[52] Trevejo-Nunez G., Elsegeiny W., Aggor F.E.Y., Tweedle J.L., Kaplan Z., Gandhi P., Castillo P., Ferguson A., Alcorn J.F., Chen K., Kolls J.K., Gaffen S.L. (2019). Interleukin-22 (IL-22) binding protein constrains IL-22 activity, host defense, and oxidative phosphorylation genes during pneumococcal pneumonia. Infect Immun.

[53] Treerat P., Prince O., Cruz-Lagunas A., Munoz-Torrico M., Salazar-Lezama M.A., Selman M., Fallert-Junecko B., Reinhardt T.A., Alcorn J.F., Kaushal D., Zuniga J., Rangel-Moreno J., Kolls J.K., Khader S.A. (2017). Novel role for IL-22 in protection during chronic Mycobacterium tuberculosis HN878 infection. Mucosal Immunol.

[54] Simonian P.L., Wehrmann F., Roark C.L., Born W.K., O'Brien R.L., Fontenot A.P. (2010). gammadelta T cells protect against lung fibrosis via IL-22. J Exp Med.

[55] Gu P., Wang D., Zhang J., Wang X., Chen Z., Gu L., Liu M., Meng F., Yang J., Cai H., Xiao Y., Chen Y., Cao M. (2021). Protective function of interleukin-22 in pulmonary fibrosis. Clin Transl Med.

[56] Qu Z., Dou W., Zhang K., Duan L., Zhou D., Yin S. (2022). IL-22 inhibits bleomycin-induced pulmonary fibrosis in association with inhibition of IL-17A in mice. Arthritis Res Ther.

[57] Liang M., Wang J., Chu H., Zhu X., He H., Liu Q., Qiu J., Zhou X., Guan M., Xue Y., Chen X., Zou H. (2013). Interleukin-22 inhibits bleomycin-induced pulmonary fibrosis. Mediators Inflamm.

[58] Lo Re S., Dumoutier L., Couillin I., Van Vyve C., Yakoub Y., Uwambayinema F., Marien B., van den Brule S., Van Snick J., Uyttenhove C., Ryffel B., Renauld J.C., Lison D., Huaux F. (2010). IL-17A-producing gammadelta T and Th17 lymphocytes mediate lung inflammation but not fibrosis in experimental silicosis. J Immunol.

[59] Andonegui G., Bonder C.S., Green F., Mullaly S.C., Zbytnuik L., Raharjo E., Kubes P. (2003). Endothelium-derived Toll-like receptor-4 is the key molecule in LPS-induced neutrophil sequestration into lungs. J Clin Invest.

[60] Taghavi S., Jackson-Weaver O., Abdullah S., Wanek A., Drury R., Packer J., Cotton-Betteridge A., Duchesne J., Pociask D., Kolls J. (2021). Interleukin-22 mitigates acute respiratory distress syndrome (ARDS). PLoS One.

[61] Martin J.C., Beriou G., Heslan M., Chauvin C., Utriainen L., Aumeunier A., Scott C.L., Mowat A., Cerovic V., Houston S.A., Leboeuf M., Hubert F.X., Hemont C., Merad M., Milling S., Josien R. (2014). Interleukin-22 binding protein (IL-22BP) is constitutively expressed by a subset of conventional dendritic cells and is strongly induced by retinoic acid. Mucosal Immunol.

[62] Lim C., Hong M., Savan R. (2016). Human IL-22 binding protein isoforms act as a rheostat for IL-22 signaling. Sci Signal.

[63] Yang H.Z., Cui B., Liu H.Z., Chen Z.R., Yan H.M., Hua F., Hu Z.W. (2009). Targeting TLR2 attenuates pulmonary inflammation and fibrosis by reversion of suppressive immune microenvironment. J Immunol.

[64] Sikic B.I. (1986). Biochemical and cellular determinants of bleomycin cytotoxicity. Cancer Surv.

[65] Chandler D.B. (1990). Possible mechanisms of bleomycin-induced fibrosis. Clin Chest Med.

[66] Ahn J., Konno H., Barber G.N. (2015). Diverse roles of STING-dependent signaling on the development of cancer. Oncogene.

[67] Kleinschmidt D., Giannou A.D., McGee H.M., Kempski J., Steglich B., Huber F.J., Ernst T.M., Shiri A.M., Wegscheid C., Tasika E., Hubener P., Huber P., Bedke T., Steffens N., Agalioti T., Fuchs T., Noll J., Lotter H., Tiegs G., Lohse A.W., Axelrod J.H., Galun E., Flavell R.A., Gagliani N., Huber S. (2017). A protective function of IL-22BP in ischemia reperfusion and acetaminophen-induced liver injury. J Immunol.

[68] Sertorio M., Hou X., Carmo R.F., Dessein H., Cabantous S., Abdelwahed M., Romano A., Albuquerque F., Vasconcelos L., Carmo T., Li J., Varoquaux A., Arnaud V., Oliveira P., Hamdoun A., He H., Adbelmaboud S., Mergani A., Zhou J., Monis A., Pereira L.B., Halfon P., Bourliere M., Parana R., Dos Reis M., Gonnelli D., Moura P., Elwali N.E., Argiro L., Li Y., Dessein A. (2015). IL-22 and IL-22 binding protein (IL-22BP) regulate fibrosis and cirrhosis in hepatitis C virus and schistosome infections. Hepatology.

[69] Braun R.K., Ferrick C., Neubauer P., Sjoding M., Sterner-Kock A., Kock M., Putney L., Ferrick D.A., Hyde D.M., Love R.B. (2008). IL-17 producing gammadelta T cells are required for a controlled inflammatory response after bleomycin-induced lung injury. Inflammation.

[70] Sandrock I., Reinhardt A., Ravens S., Binz C., Wilharm A., Martins J., Oberdorfer L., Tan L., Lienenklaus S., Zhang B., Naumann R., Zhuang Y., Krueger A., Forster R., Prinz I. (2018). Genetic models reveal origin, persistence and non-redundant functions of IL-17-producing gammadelta T cells. J Exp Med.

[71] Jameson J.M. (2018). Gammadelta T cells: a disappearing act with a big reveal. J Exp Med.

[72] Prinz I., Silva-Santos B., Pennington D.J. (2013). Functional development of gammadelta T cells. Eur J Immunol.

[73] Heilig J.S., Tonegawa S. (1986). Diversity of murine gamma genes and expression in fetal and adult T lymphocytes. Nature.

[74] Wadia P., Atre N., Pradhan T., Mistry R., Chiplunkar S. (2005). Heat shock protein induced TCR gammadelta gene rearrangements in patients with oral cancer. Oral Oncol.

[75] Adams E.J., Strop P., Shin S., Chien Y.H., Garcia K.C. (2008). An autonomous CDR3delta is sufficient for recognition of the nonclassical MHC class I molecules T10 and T22 by gammadelta T cells. Nat Immunol.

[76] Leslie D.S., Vincent M.S., Spada F.M., Das H., Sugita M., Morita C.T., Brenner M.B. (2002). CD1-mediated gamma/delta T cell maturation of dendritic cells. J Exp Med.

[77] Zhang H., Hu H., Jiang X., He H., Cui L., He W. (2005). Membrane HSP70: the molecule triggering gammadelta T cells in the early stage of tumorigenesis. Immunol Invest.

