# Supplementary figures and images for: IL-22 Binding Protein Controls IL-22–Driven Bleomycin-Induced Lung Injury
Source: Am J Pathol. 2024 Mar;194(3):338–52. doi: 10.1016/j.ajpath.2023.11.011 (PMC10913761; doi:10.1016/j.ajpath.2023.11.011)

Supplemental Figure 1.

A.

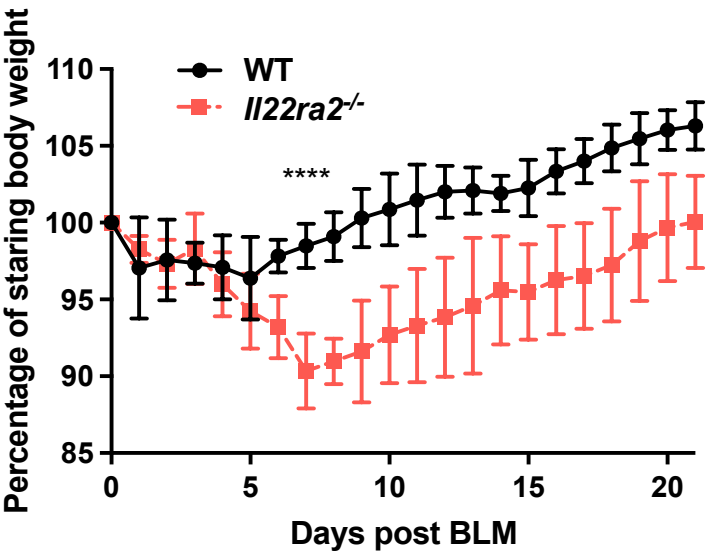

B.

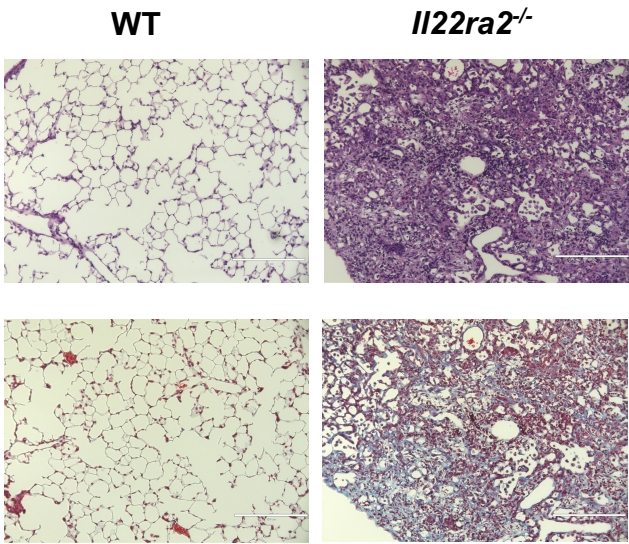

C.

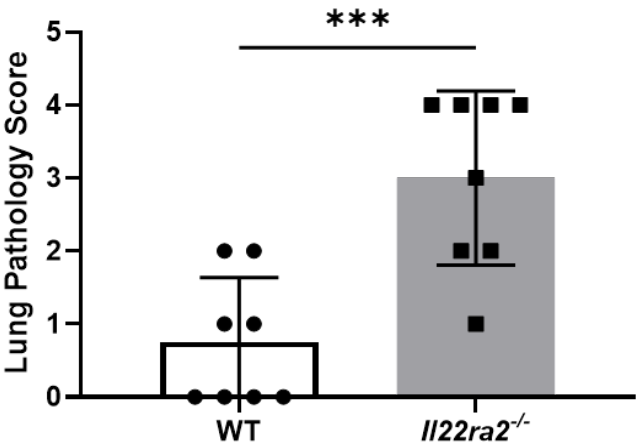

Supplement: Supplemental Figure S1 — Il22ra2−/− mice show higher morbidity and more lung injury than wild-type (WT) mice on day 21 after bleomycin treatment. WT and Il22ra2−/− mice ages 6 to 8 weeks were treated with bleomycin at a lower dose (0.0025 U/g) intratracheally. A: Morbidity was measured by weight loss. B: Histology of the lungs from both groups on day 21 measured by hematoxylin and eosin (H&E) staining and Masson's trichrome staining. C: Lung pathology was scored blindly as described in Materials and Methods. n = 6 mice/group (A); n = 8 mice/group (C). ∗∗∗P < 0.001, unpaired t-test (C); ∗∗∗∗P < 0.0001, ordinary two-way analysis of variance (A). Scale bars = 200 μm. BLM, bleomycin. [file mmc1.pdf]

## Supplemental Figure 2.

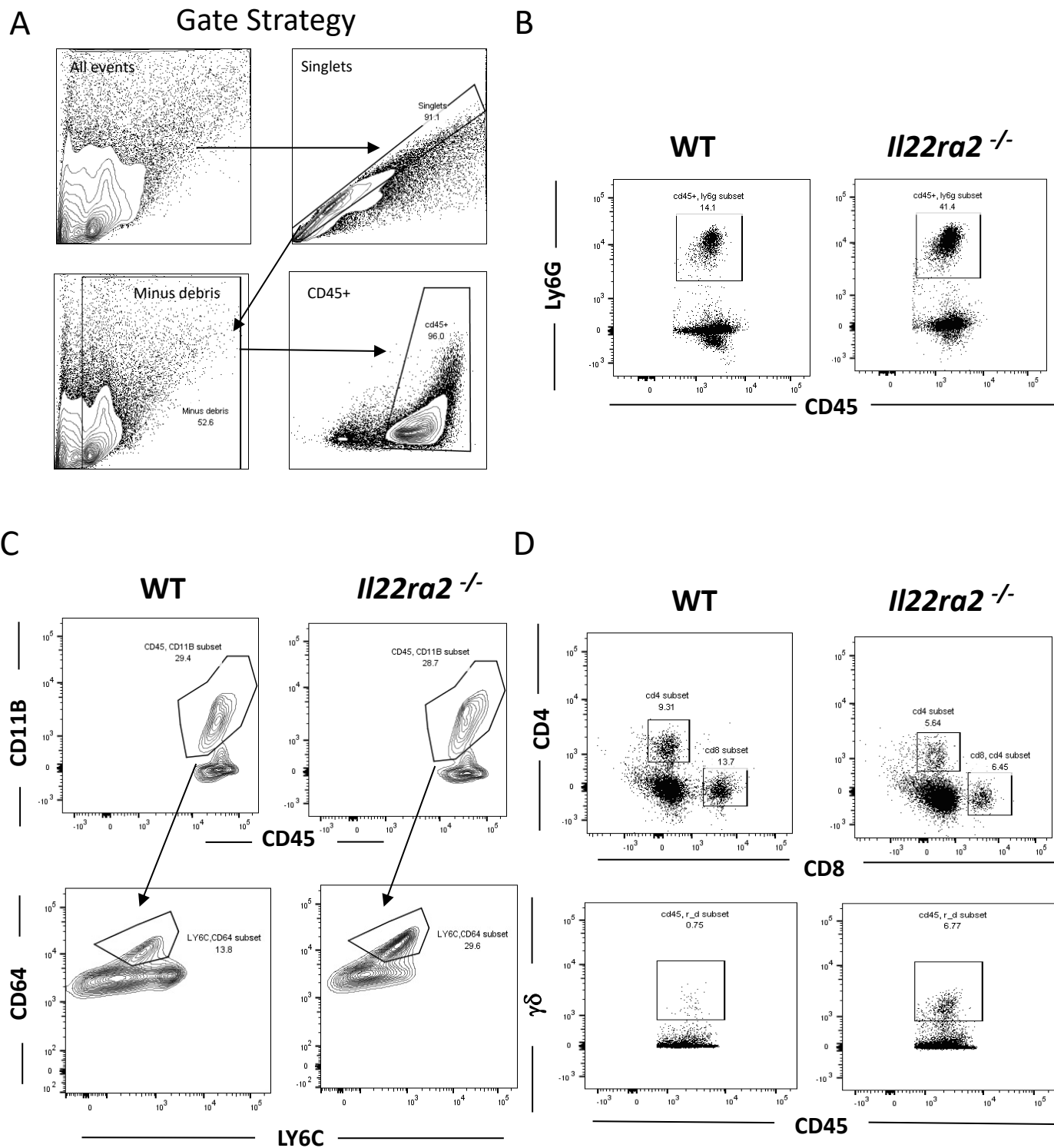

Supplement: Supplemental Figure S2 — More γδ T cells, neutrophils, and inflammatory monocytes in the lungs of Il22ra2−/− mice than in wild-type (WT) mice after bleomycin treatment. WT and Il22ra2−/− mice ages 6 to 8 weeks were treated with bleomycin (0.003 U/g) intratracheally. Mice were euthanized on day 4. Lungs were collected and went through single-cell preparation. A: Gating strategy deployed to show all events, singlets, minus debris, and CD45+ populations. B: Flow cytometry representative dot plots showed CD45+Ly6g+ (neutrophils). C: CD45+CD64+CD11B+Ly6C+ (inflammatory monocytes). D: CD4 T cells and γδ T cells. [file mmc2.pdf]

Supplemental Figure 3.

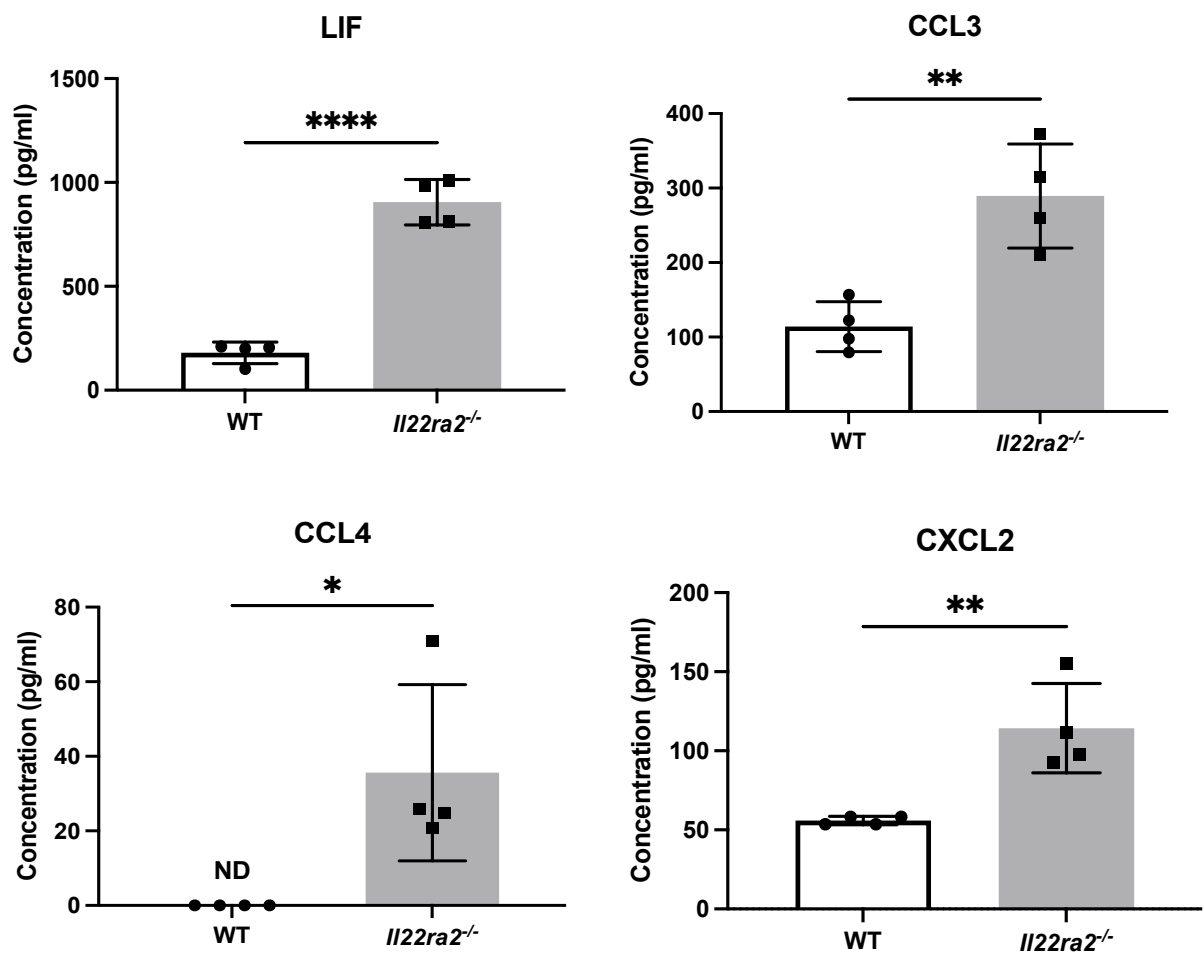

Supplement: Supplemental Figure S3 — Higher inflammatory cytokines and chemokines in the Il22ra2−/− mice after bleomycin treatment. Wild-type (WT) and Il22ra2−/− mice ages 6 to 8 weeks were treated with bleomycin (0.003 U/g) intratracheally. Mice were euthanized on day 4. Protein was isolated from whole lungs. Leukemia inhibitory factor (LIF), CCL3, CCL4, and CXCL2 protein levels were detected by Bio-Plex. n = 4 mice/group. ∗P < 0.05, ∗∗P < 0.01, and ∗∗∗∗P < 0.0001, unpaired t-test. ND, none determined. [file mmc3.pdf]

Supplemental Figure 4.

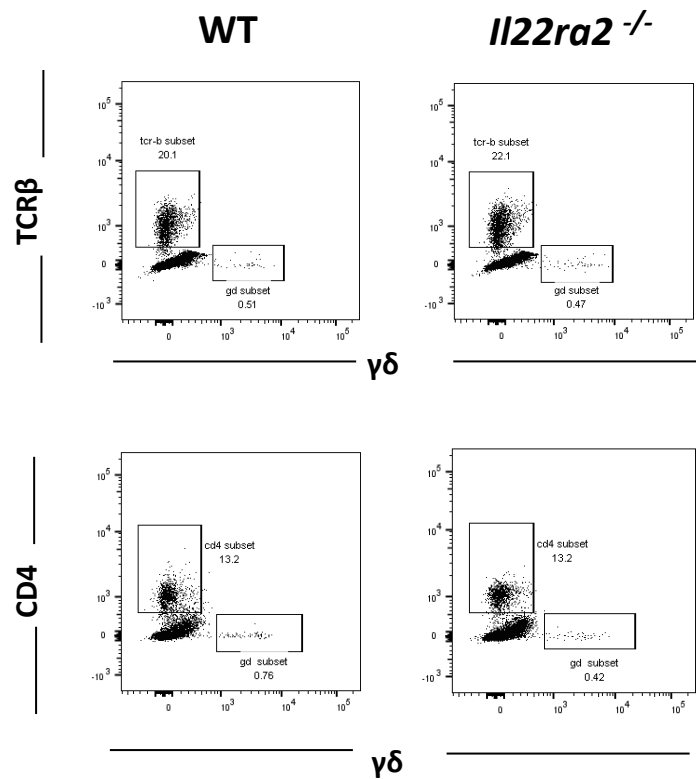

Supplement: Supplemental Figure S4 — No difference in the CD4 and γδ T-cell populations between naïve WT and Il22ra2−/− mice. Naïve wild-type and Il22ra2−/− mice ages 6 to 8 weeks were used. Lungs were collected and went through single-cell preparation. Flow cytometry representative dot plots showed CD45+ cells, gated on T-cell receptor beta (TCRβ) versus γδ receptor and CD4 versus γδ receptor. [file mmc4.pdf]

Supplemental Figure 5.

A

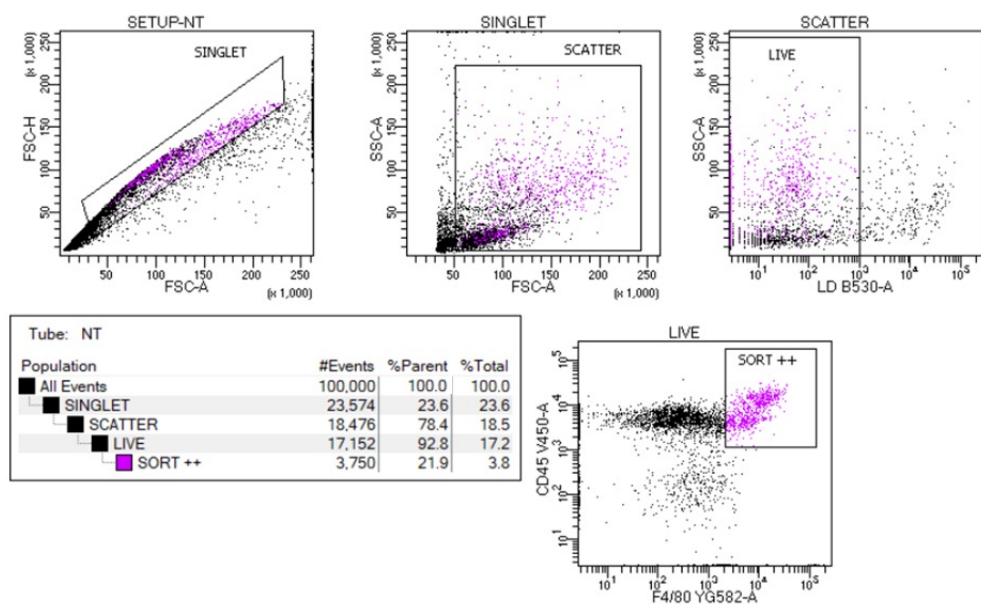

B

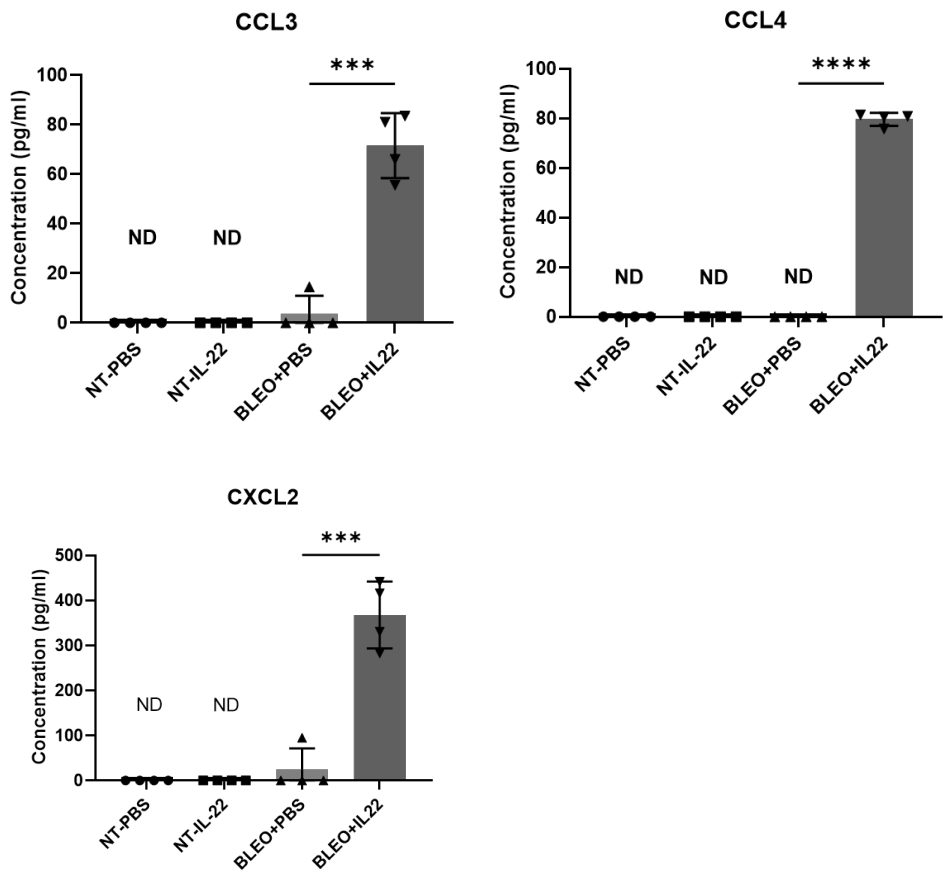

Supplement: Supplemental Figure S5 — Macrophages sorted from bleomycin-treated wild-type (WT) mice lungs secrete more inflammatory cytokines and chemokines after IL-22 activation. Flow cytometry cell sorting was performed on the lungs from bleomycin not treated (NT) and bleomycin-treated WT mice. The CD45+F4/80+ population was sorted and treated with phosphate-buffered saline (PBS) and IL-22 (30 ng/well) in vitro. A: Sorting strategy for the CD45+F4/80+ population. Cells first were gated on singlet, then scatter and live cells, and last on CD45+F4/80+. B: CCL3, CCL4, and CXCL2 protein levels detected from the cell culture supernatant by Bio-Plex. n = 4 mice/group. ∗∗∗P < 0.001, ∗∗∗∗P < 0.0001, unpaired t-test. BLEO, bleomycin; FSC-A, forward scatter area; FSC-H, forward scatter height; ND, not detected; SSC-A, side scatter area. [file mmc5.pdf]

Supplemental Figure 6.

A

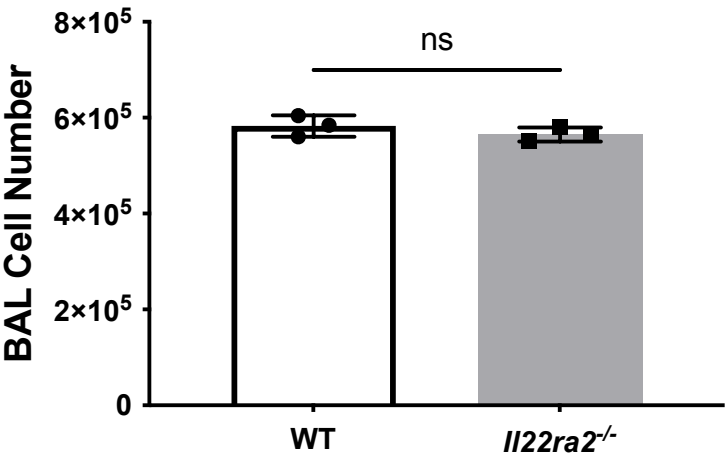

B

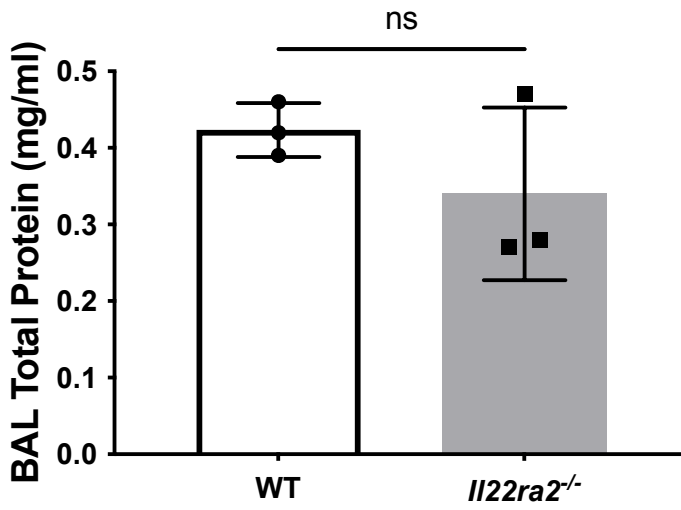

C

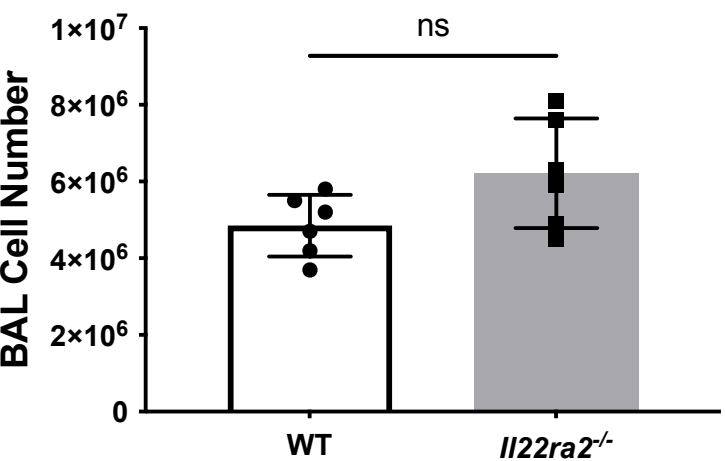

D

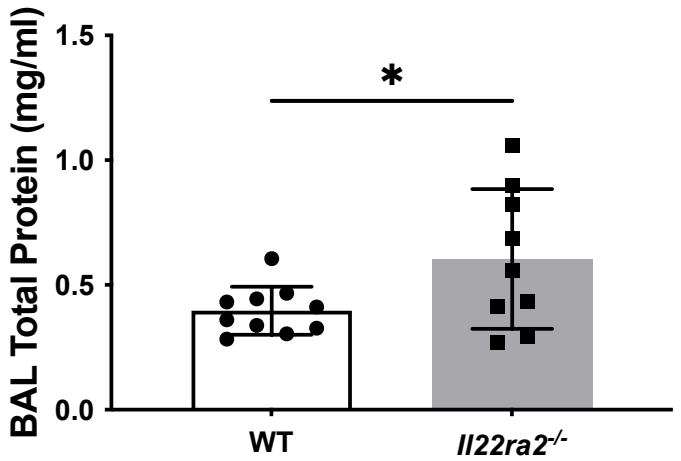

Supplement: Supplemental Figure S6 — Lipopolysaccharide (LPS) challenge of wild-type (WT) and Il22ra2−/− mice show no differences in initial (48 hours) response, but delayed recovery of inflammation in Il22ra2−/− mice. LPS (50 μg in 50 μL phosphate-buffered saline) was administered by tracheal aspiration in 6- to 8-week-old male WT and Il22ra2−/− mice. There were no differences in weight loss or recovery in these mice. Mice were euthanized on days 2 and 4 after administration. A and B: Lungs were lavaged and analysis of bronchoalveolar lavage (BAL) showed no differences in lung (A) BAL cell number or (B) total BAL protein 48 hours after administration. C and D: Four days after administration, mild but significant delayed clearance of (C) BAL cell number and (D) total BAL protein was shown. ∗P < 0.05 as determined by unpaired t-test. ns, not significant. [file mmc6.pdf]
